# Long‐Term Ambient Benzene Exposure and Brain Disorders Among Urban Adults: Effect Modification by Genetic Susceptibility and Potential Mediation by Plasma Proteins

**DOI:** 10.1002/advs.75874

**Published:** 2026-05-29

**Authors:** Jianhui Guo, Xia Zhong, Petros Koutrakis, Carolina L. Zilli Vieira, Feifei Si, Yaqi Wang, Xinyao Lian, Yi Fan, Zhaokun Wang, Shaodan Huang, Jing Li

**Affiliations:** ^1^ Institute of Child and Adolescent Health School of Public Health National Health Commission Key Laboratory of Reproductive Health Peking University Beijing China; ^2^ Department of Environmental Health Harvard T.H. Chan School of Public Health Boston USA; ^3^ Peking University Sixth Hospital/Institute of Mental Health NHC Key Laboratory of Mental Health (Peking University) National Clinical Research Center for Mental Disorders (Peking University Sixth Hospital) Beijing China; ^4^ Department of Occupational and Environmental Health Sciences School of Public Health Peking University Beijing China; ^5^ Key Laboratory of Epidemiology of Major Diseases (Peking University) Ministry of Education Beijing China

## Abstract

Benzene is a common urban air pollutant with potential neurotoxicity, yet the health effects of long‐term, low‐level ambient exposure and the underlying mechanisms remain poorly characterized. Here, we used data from 288,180 UK Biobank (UKB) participants to investigate associations between benzene exposure and brain disorders as well as related genetic susceptibility and potential protein‐mediated mechanisms. We found that benzene exposure was significantly associated with seven brain disorders, including anxiety disorders, dementia, epilepsy, major depressive disorder, migraine, sleep disorders, and schizophrenia spectrum disorders. Greater genetic susceptibility to benzene exposure was most apparent for epilepsy, migraine, sleep disorder, and schizophrenia spectrum disorders. Pathway‐level mediation analyses identified 40 pathways that may mediate the associations between benzene exposure and brain disorders, converging on pathways regulating cell survival and apoptosis, immune‐inflammatory signaling, cellular stress responses, and cell–cell or cell–matrix interactions, highlighting coordinated disruptions in cellular homeostasis and immune regulation. Overall, our findings suggest that even low‐level ambient benzene exposure may be linked to adverse brain health through multiple potential biological pathways, highlighting the need for further studies in diverse exposure settings, including rural populations with potentially higher benzene exposure.

## Graphical abstract

Low‐level ambient benzene exposure is associated with increased risks of multiple brain disorders in urban adults. Genetic susceptibility modifies these associations, while plasma proteomics points to potential biological pathways linking benzene exposure to adverse brain health. The findings highlight the neuropsychiatric burden of ambient benzene at relatively low exposure levels.

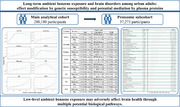

## 1 Introduction

Brain disorders, including neurological and psychiatric conditions, are major contributors to the global burden of disease and represent critical targets for advancing sustainable development [1, 2]. As populations increasingly concentrate in urban areas worldwide, attention has shifted toward understanding how urbanization shapes both physical and mental health [3]. Emerging evidence suggests that distinct urban environmental features may influence specific clusters of psychiatric symptoms through unique neurobiological pathways [4]. Within this context, ambient air pollutants routinely encountered by urban residents have gained prominence as important and modifiable environmental risk factors [5].

Benzene is among the most widespread volatile organic compounds in the atmosphere, arising from various anthropogenic sources, including industrial activities, motor vehicle emissions, fuel evaporation, solvent use, and biomass burning [6, 7]. As a result, benzene persists in urban environments and represents a vital air pollutant associated with long‐term, low‐level exposure among urban populations [8, 9]. Benzene is a well‐established human carcinogen and has been linked to a spectrum of adverse health outcomes across both short‐ and long‐term exposure windows [6, 10, 11, 12]. No evidence supports the existence of a safe threshold for benzene exposure [10]. Emerging studies indicate that even low ambient concentrations may confer measurable health risks [10, 11, 13, 14]. By contrast, epidemiological evidence remains extremely limited, with only one study to date having evaluated the effects of long‐term, environmental‐level benzene exposure on brain health among urban residents [11].

Moreover, evidence on the neurotoxic mechanisms of benzene has been derived primarily from animal studies and occupational exposure cohorts [15, 16, 17, 18], whereas only one study to date has examined the neurobiological effects of low‐level ambient benzene exposure on brain disorders in the general population [11]. Robust population‐based evidence is therefore needed to elucidate the mechanisms through which long‐term, low‐level ambient benzene exposure may contribute to neurotoxicity. First, brain disorders show substantial genetic susceptibility, highlighting the need to clarify whether benzene contributes to disease development through interactions with polygenic predisposition, an area in which evidence remains scarce [19]. Besides, elucidating benzene‐induced changes in biomarkers is essential for understanding its potential neurotoxic mechanisms, and proteomics provides a practical approach for systematically characterizing the associated functional biological perturbations [20]. However, existing omics‐based studies of environmental pollutant toxicity frequently rely on reductionist, bottom‐up analytic frameworks that evaluate individual biomarkers in isolation before integrating them through bioinformatic analyses [21]. Such approaches have limited capacity to capture synergistic, antagonistic, or compensatory interactions among molecules within the same biological pathway [22]. These limitations underscore the need for a multidimensional mediation framework capable of evaluating both single‐protein effects and coordinated pathway‐level regulation, thereby providing a more comprehensive understanding of the biological mechanisms linking ambient benzene exposure to brain health [23].

**FIGURE 1:**
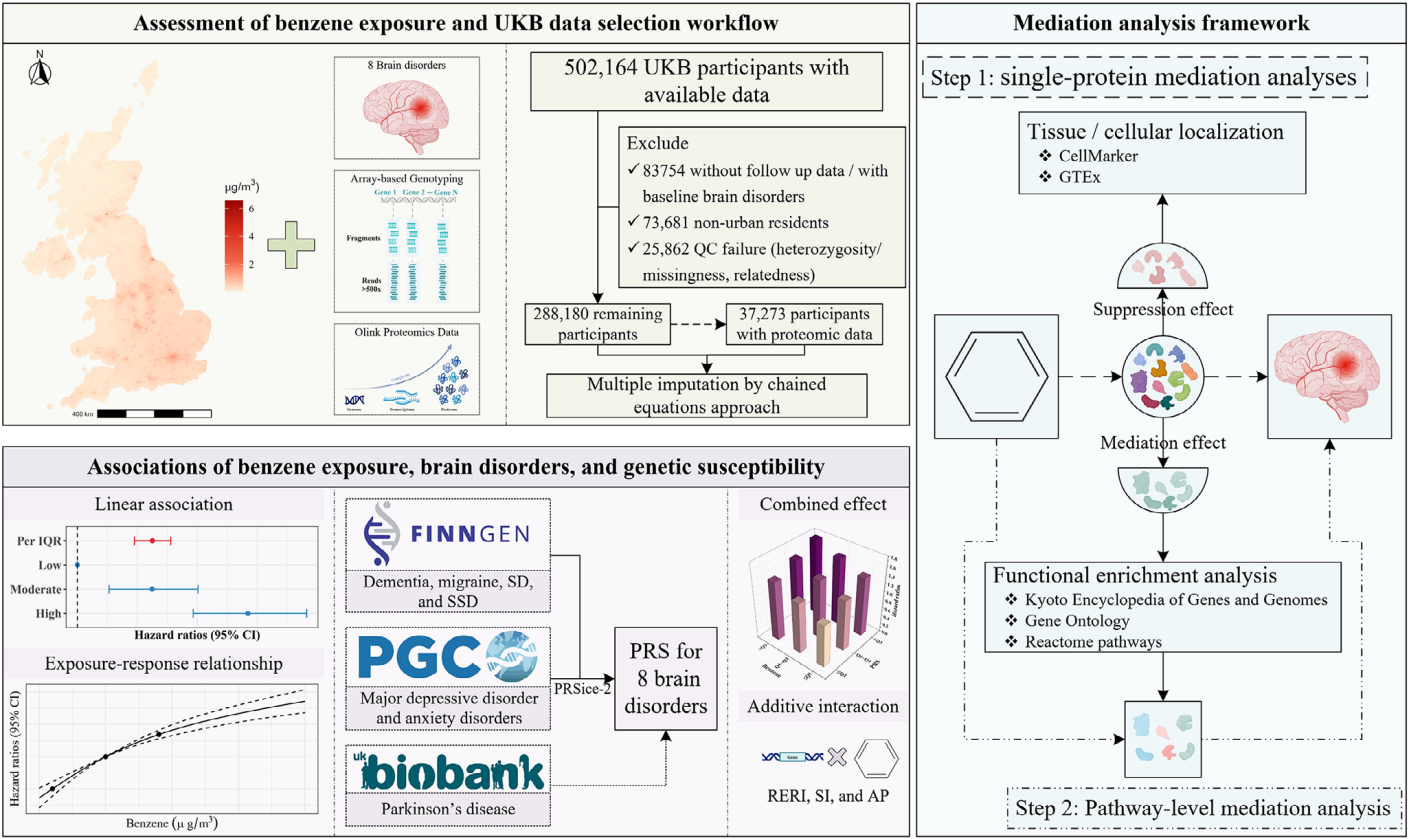
Overview of analytic design. The flowchart was created using BioRender (Agreement number: NP291YV20W; https://www.biorender.com/). SD, Sleep disorders; SSD, schizophrenia spectrum disorders; PRS, polygenic risk scores; RERI, relative excess risk due to interaction; SI, synergy index; AP, attributable proportion due to interaction.

To address these knowledge gaps, we used multimodal data from UK Biobank (UKB) participants to systematically examine the association between long‐term, low‐level ambient benzene exposure and eight brain disorders (Figure 1). We first examined the linear association and the exposure‐response relationship between ambient benzene exposure and brain disorders. We then constructed polygenic risk scores (PRS) for each disorder to evaluate the joint and interactive effects of benzene exposure and genetic susceptibility on brain disorders. We also developed a multidimensional mediation analysis framework that enables a comprehensive evaluation of the potential mediating roles of proteins and their associated biological pathways in the association between benzene exposure and brain disorders. Our study aims to deepen understanding of the potential mechanisms by which benzene exposure contributes to brain disorders by synthesizing the reliable evidence and to provide scientific support for the management of ambient benzene, the optimization of air quality standards, and urban health planning and sustainable development.

## 2 Results

A total of 288,180 participants were included in this study (Table S7), with 48.0% being male and a mean baseline age of 56.5 years (standard deviation 8.2). Over a mean follow‐up of 14.0 years (standard deviation 2.3), 12,329 individuals (4.3%) developed incident anxiety disorders, 4,897 (1.7%) developed dementia, 2,003 (0.7%) developed epilepsy, 12,319 (4.3%) developed major depressive disorder (MDD), 3,378 (1.2%) developed migraine, 1,955 (0.7%) developed Parkinson's disease (PD), 5,893 (2.0%) developed sleep disorders (SD), and 556 (0.2%) developed schizophrenia spectrum disorders (SSD). The mean age at diagnosis for each condition is provided in Table S7. Between 2003 and 2010, benzene concentrations in the United Kingdom generally declined, whereas their spatial distribution remained essentially unchanged. Elevated concentrations were mainly observed in south‐central England and around major urban areas and transport corridors (Figure S2). At baseline (Table S7), the mean benzene exposure level among all participants was 0.72 µg/m^3^ (standard deviation 0.20).

**FIGURE 2:**
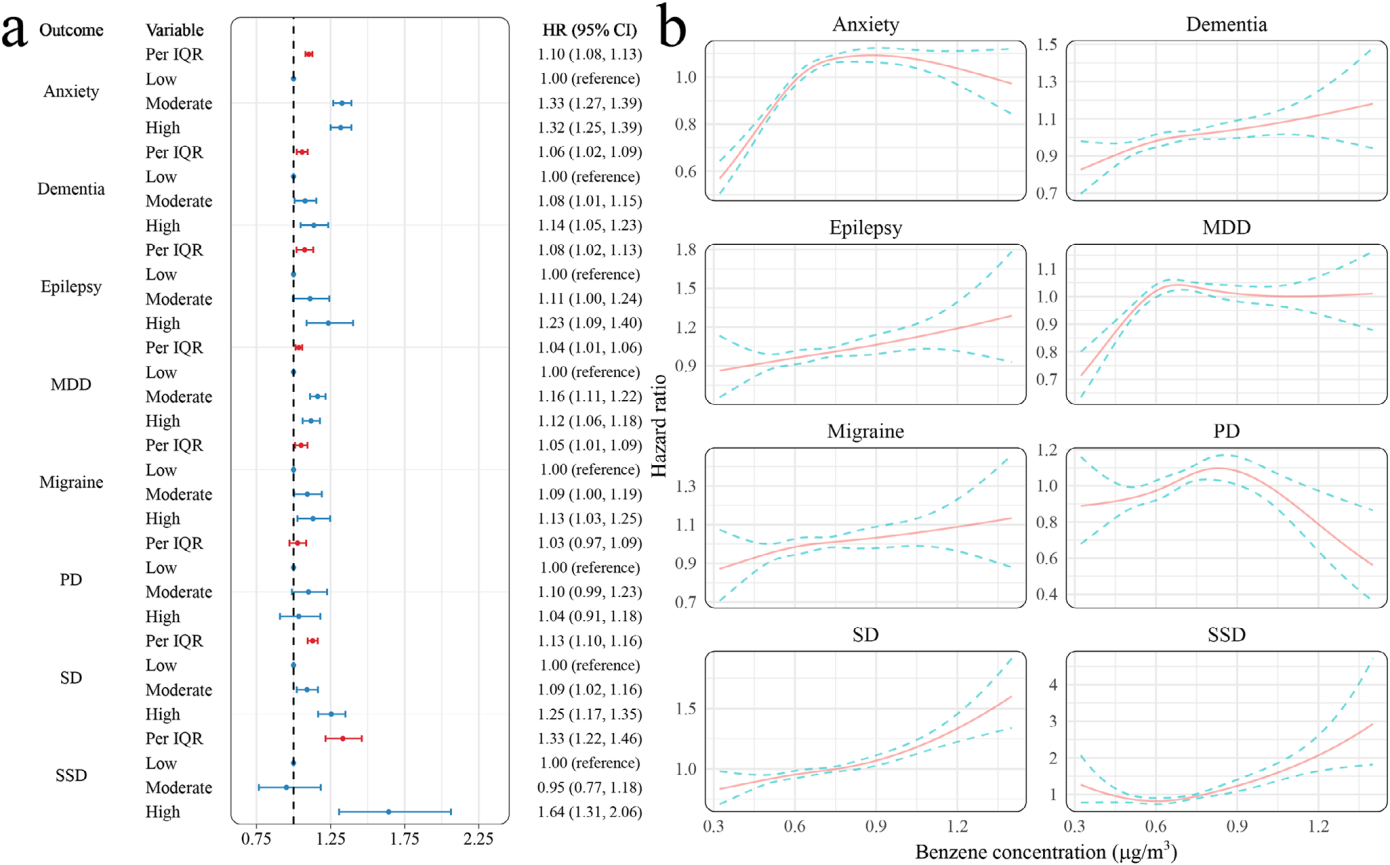
Association between ambient benzene exposure and incident brain disorders. (a) Hazard ratios were estimated using Cox proportional hazards regression. Benzene exposure was categorized into three increasing levels according to the first and third quartiles: low (≤0.58 µg/m^3^), moderate (0.58–0.82 µg/m^3^), and high (>0.82 µg/m^3^). (**b)** Exposure‐response relationships were estimated using natural cubic spline regression with knots placed at the 25^th^, 50^th^, and 75^th^ percentiles of the benzene exposure distribution. Two‐sided *Z*‐tests were used to assess statistical significance. All analyses were adjusted for age, sex, qualifications, BMI, Charlson Comorbidity Index (CCI), occupational benzene exposure, alcohol consumption, smoking history, moderate sleep, moderate physical activity, healthy diet score, proximity to major roads, and noise pollution. MDD, major depressive disorder; PD, Parkinson's disease; SD, Sleep disorders; SSD, schizophrenia spectrum disorders.

Significant associations were observed between benzene exposure and the risk of seven brain disorders in Cox proportional hazards models after adjustment for potential confounders (Figure 2A and Table S8). We found that a 0.24 µg/m^3^ increase in the interquartile range (IQR) of benzene exposure was associated with a 10% [95% confidence interval (CI): 1.08, 1.13, *p* = 3.09 × 10^−20^] higher risk of anxiety disorders. Similar positive associations were observed for dementia [Hazard ratios (HR) = 1.06; 95% CI: 1.02, 1.09; *p* = 1.47 × 10^−03^], epilepsy (HR = 1.08; 95% CI: 1.02, 1.13; *p* = 6.72 × 10^−03^), MDD (HR = 1.04; 95% CI: 1.01, 1.06; *p* = 1.22 × 10^−03^), migraine (HR = 1.05; 95% CI: 1.01, 1.09; *p* = 1.44 × 10^−02^), SD (HR = 1.13; 95% CI: 1.10, 1.16; *p* = 2.55 × 10^−15^), and SSD (HR = 1.33; 95% CI: 1.22, 1.46; *p* = 7.69 × 10^−10^). Figure 2A also shows the results from incorporating IQR‐defined three‐level categories of continuous benzene exposure into the Cox proportional hazards regression analysis, yielding estimates that closely align with the exposure‐response curves. For anxiety disorders and MDD, the risk increased from the lowest to the middle exposure group and then plateaued. Similar monotonic increasing trends were demonstrated by dementia, epilepsy, migraine, SSD, and SD, although the pattern for SSD was not strictly monotonic across exposure categories. In contrast, the association for PD followed an inverted‐V‐shaped pattern, with the highest risk observed in the middle exposure group (Figure 2B). The associations between ambient benzene exposure and brain disorders across subgroups are shown in Figure S3. Additionally, when further adjusting the fully adjusted models for PM_2.5_, NO_2_, and NOx, the associations between ambient benzene exposure and most brain disorders remained robust (Figure S4). The results from the time‐dependent Cox regression models were generally consistent with those from the Cox proportional hazards models, and no obvious lag effect was observed (Table S9).

**FIGURE 3:**
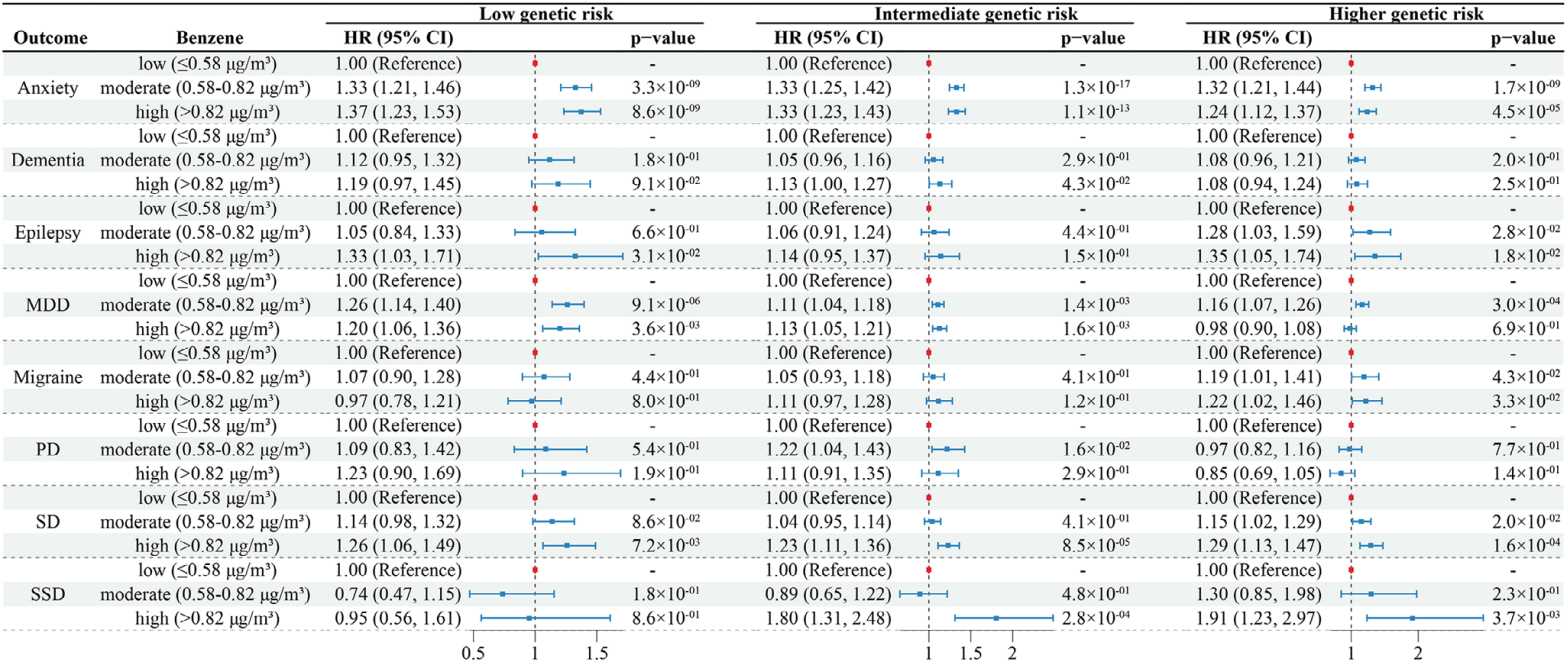
Risk of incident brain disorders according to categories of benzene exposure within each genetic risk stratum. Benzene exposure was categorized into three increasing levels according to the first and third quartiles: low (≤0.58 µg/m^3^), moderate (0.58–0.82 µg/m^3^), and high (>0.82 µg/m^3^). Genetic risk was classified into lower, intermediate, and higher groups according to the distribution of the polygenic risk score (PRS): lower (≤Q1), intermediate (Q1–Q3), and higher (>Q3). Hazard ratios (HRs) were estimated using Cox proportional hazards models with the low benzene group as the reference, adjusted for age, sex, qualifications, BMI, Charlson Comorbidity Index (CCI), occupational benzene exposure, alcohol consumption, smoking history, moderate sleep, moderate physical activity, healthy diet score, proximity to major roads, and noise pollution. MDD, major depressive disorder; PD, Parkinson's disease; SD, Sleep disorders; SSD, schizophrenia spectrum disorders. Two‐sided *Z*‐tests were used to assess statistical significance. *N*, number of individuals at risk; *n*, number of cases.

After stratification by genetic risk (Figure 3), the associations between benzene exposure and neuropsychiatric outcomes were heterogeneous, whereas the association with anxiety remained consistent across strata. In the low‐, intermediate‐, and high‐genetic‐risk groups, the HRs (95% CI) for moderate exposure were 1.33 (1.21, 1.46), 1.33 (1.25, 1.42) and 1.32 (1.21, 1.44), respectively; the corresponding HRs (95% CI) for high exposure were 1.37 (1.23, 1.53), 1.33 (1.23, 1.43), and 1.24 (1.12, 1.37). MDD showed a similar pattern, with moderate exposure associated with increased risk in all three genetic‐risk groups. By contrast, the associations for epilepsy and SSD were more evident in individuals at higher genetic risk. In the high‐genetic‐risk group, the HRs (95% CI) for epilepsy were 1.28 (1.03, 1.59) for moderate exposure and 1.35 (1.05, 1.74) for high exposure. For SSD, the HRs (95% CI) under high exposure were 1.80 (1.31, 2.48) and 1.91 (1.23, 2.97) in the intermediate‐ and high‐genetic‐risk groups, respectively. Together, these data indicate that the association between benzene exposure and anxiety is largely consistent across genetic‐risk strata, whereas the excess risks of epilepsy and SSD are more apparent among those with greater genetic susceptibility. The joint associations of benzene exposure and genetic risk showed that individuals with both high benzene exposure and high genetic risk had the highest incidence risks for several brain disorders, particularly epilepsy, migraine, SD, and SSD (Table S10). Overall, no clear additive interaction was detected in the gene–environment interaction analysis (Table S11).

**FIGURE 4:**
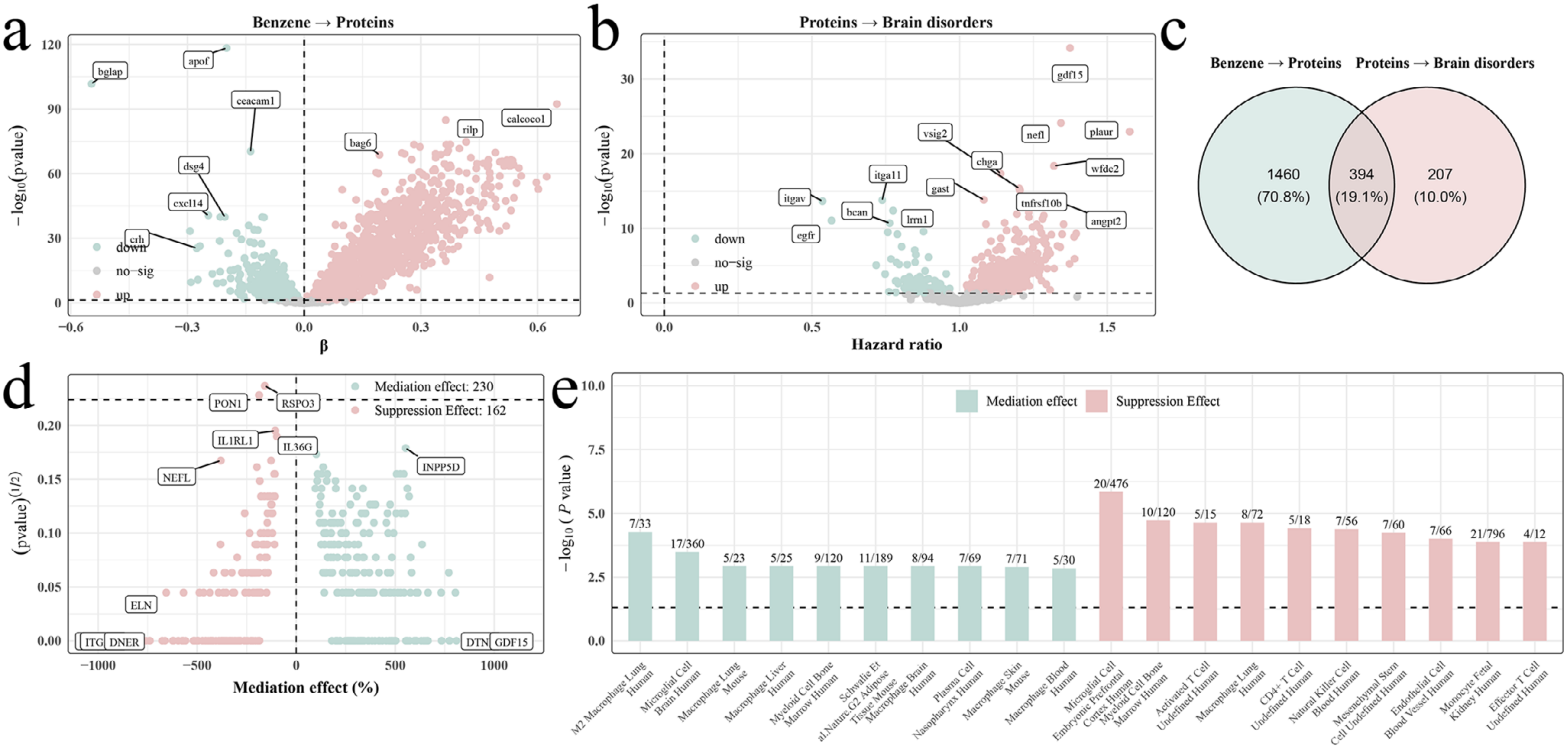
Mediation analysis results for proteins in the association between benzene exposure and brain disorders. (a) Associations between benzene exposure and proteins were estimated using linear regression models. (**b)** Associations between proteins and the risk of brain disorders were estimated using Cox proportional hazards models. (**c)** The overlap between proteins that were statistically significant in panels a and b was identified. (**d)** Mediation analysis was performed to quantify the proportion of benzene‐related brain disorder risk mediated by each protein. (**e)** Cell‐type enrichment analysis using CellMarker for proteins with positive mediation effects and proteins with suppression effects. The bar plot shows the top 10 significantly enriched cell types for each protein set, highlighting distinct enrichment patterns between the two groups. All analyses were adjusted for age, sex, qualifications, BMI, Charlson Comorbidity Index (CCI), occupational benzene exposure, alcohol consumption, smoking history, moderate sleep, moderate physical activity, healthy diet score, proximity to major roads, and noise pollution. Statistical significance was assessed using two‐sided *Z*‐tests.

Using a linear regression model (Figure 4A), we identified 1,854 proteins significantly associated with benzene exposure (BH‐adjusted *p* < 0.05). A Cox proportional hazards model (Figure 4B) further identified 601 proteins associated with the risk of brain disorders (BH‐adjusted *p* < 0.05). Intersecting these two sets yielded 394 proteins, which were then identified as candidate mediators (Figure 4C). Bioinformatic analyses identified significant enrichment of 40 Kyoto Encyclopedia of Genes and Genomes (KEGG) pathways and 264 Gene Ontology (GO) Biological Process terms (adjusted *p* < 0.05), with comprehensive pathway‐ and protein‐level findings detailed in Figure S5. Mediation analysis (Figure 4D and Table S12) identified 230 proteins that were considered putative mediators of the association between benzene exposure and risk of brain disorders. In contrast, the remaining 162 proteins exhibited suppression effects. Tissue localization profiling using CellMarker (Figure 4E) and independent validation in GTEx revealed an evident dichotomy between the two protein sets. CellMarker annotation suggested distinct cell‐type enrichment patterns between the two protein sets. Proteins identified as putative mediators of the association between benzene exposure and brain disorders were predominantly enriched in macrophage‐, microglia‐, and other myeloid‐related cell types. In contrast, proteins with suppressive effects showed a broader and more heterogeneous enrichment pattern involving both myeloid and lymphoid immune cell annotations, including activated T cells, CD4+ T cells, regulatory T cells, natural killer cells, monocytes, and macrophages. GTEx analysis further supported distinct tissue‐expression contexts between the two sets: putative mediators were more frequently enriched in spleen‐, liver‐, and adipose‐related signatures, whereas proteins with suppressive effects were more often associated with whole blood, lung, and vascular‐related signatures (Table S13).

**FIGURE 5:**
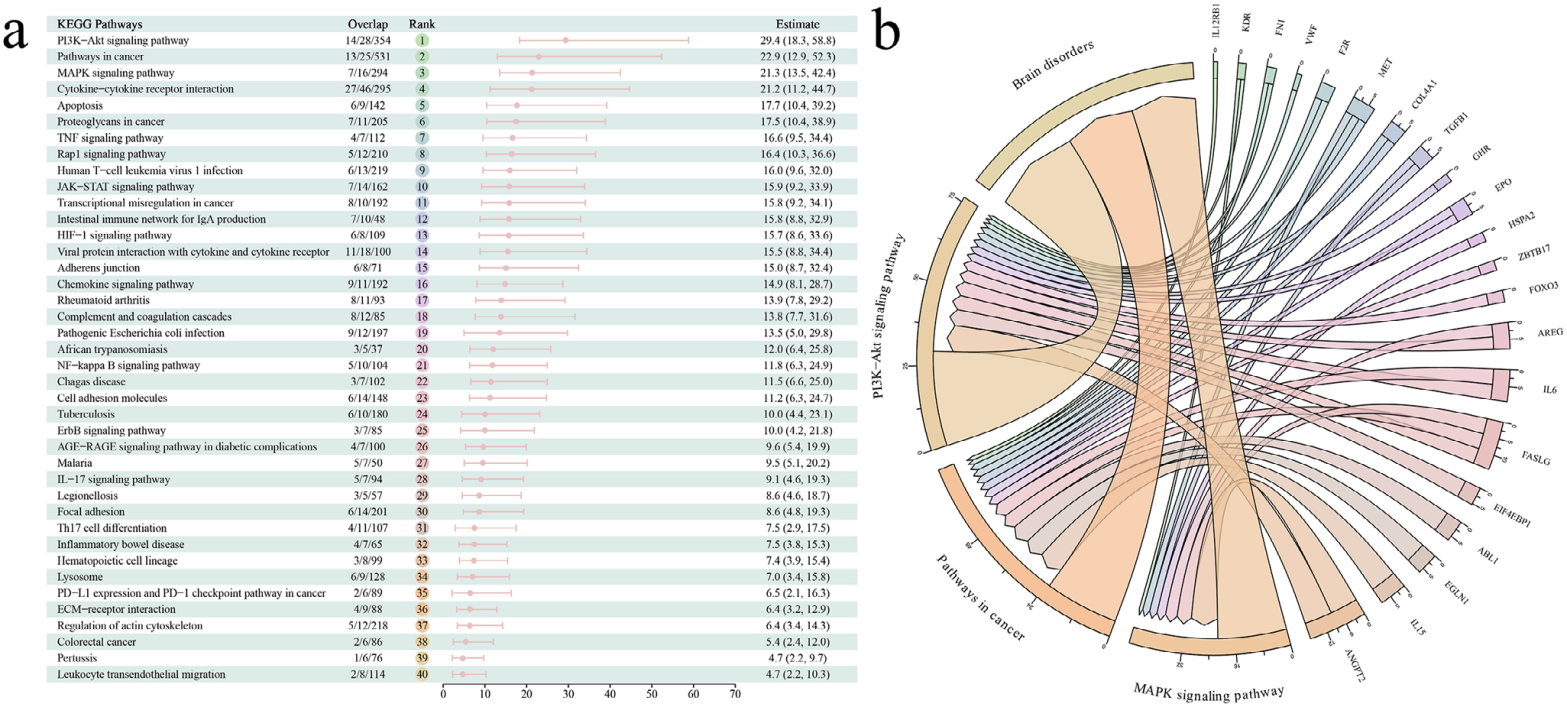
Pathway‐level mediation analysis of Kyoto Encyclopedia of Genes and Genomes (KEGG) pathways based on positively mediating proteins. (a) Pathway‐level mediation proportions were estimated by jointly modelling all positively mediating proteins within each pathway. Overlap is expressed as *a/b/c*, where *c* is the total number of pathway‐annotated proteins in KEGG, *b* is the number of analyzed proteins mapping to the pathway, and *a* is the subset of those proteins showing positive mediation effects and therefore included in the joint pathway‐level mediation model. Rank presents the ordering of pathways by mediation proportion. (b) Chord diagram summarizing mediation effects from individual proteins to their corresponding pathways and, subsequently, to brain disorders. The thickness of each link represents the magnitude of the mediation effect. Only the top three pathways with the highest mediation proportions are shown. All analyses were adjusted for age, sex, qualifications, BMI, Charlson Comorbidity Index (CCI), occupational benzene exposure, alcohol consumption, smoking history, moderate sleep, moderate physical activity, healthy diet score, proximity to major roads, and noise pollution. Statistical significance was assessed using two‐sided *Z*‐tests.

**FIGURE 6:**
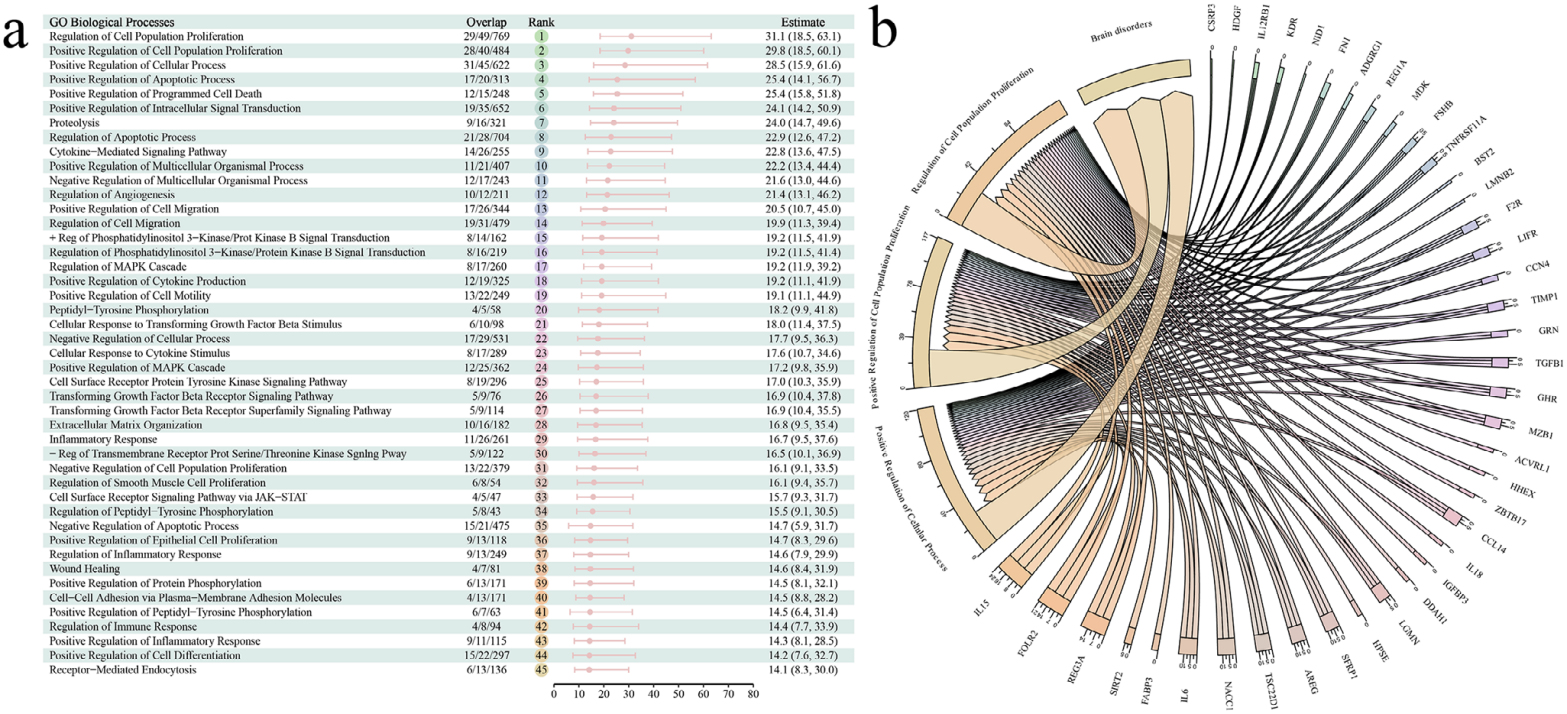
Pathway‐level mediation analysis of Gene Ontology (GO) Biological Processes based on positively mediating proteins. (a) Pathway‐level mediation proportions were estimated by jointly modeling all positively mediating proteins within each GO Biological Process. Only the top 45 of the 253 terms ranked by mediation proportion are shown. Overlap is expressed as *a/b/c*, where *c* is the total number of pathway‐annotated proteins in GO Biological Process, *b* is the number of analyzed proteins mapping to the pathway, and *a* is the subset of those proteins showing positive mediation effects and therefore included in the pathway‐level mediation model. Rank presents the ordering of pathways by mediation proportion. (b) Chord diagram summarizing mediation effects from individual proteins to their corresponding pathways and, subsequently, to brain disorders. The thickness of each link represents the magnitude of the mediation effect. Only the top three terms with the highest mediation proportions are shown. All analyses were adjusted for age, sex, qualifications, BMI, Charlson Comorbidity Index (CCI), occupational benzene exposure, alcohol consumption, smoking history, moderate sleep, moderate physical activity, healthy diet score, proximity to major roads, and noise pollution. Statistical significance was assessed using two‐sided *Z*‐tests.

We further performed pathway‐level mediation analyses. After removing proteins that exhibited suppression effects, all 40 KEGG pathways showed significant mediating roles in the association between benzene exposure and brain disorders, with mediation proportions ranging from 4.7% to 29.4% (Figure 5A and Table S14). The pathways with the largest mediation effects were the PI3K‐Akt signaling pathway (29.4%, 95% CI: 18.3, 58.8), pathways in cancer (22.9%, 95% CI: 12.9, 52.3), and the MAPK signaling pathway (21.3%, 95% CI: 13.5, 42.4). The key proteins involved in these three pathways primarily include ANGPT2, IL15, ABL1, EGLN1, EIF4EBP1, and FASLG (Figure 5B). At the GO Biological Process level, mediation analyses were conducted across 264 terms. After removing proteins with suppression effects, 253 processes significantly mediated the associations between benzene exposure and brain disorders, with mediation proportions ranging from 1.24% to 35.47% (Figure 6A and Table S15). The processes with the largest mediation effects were regulation of cell population proliferation (31.1%, 95% CI: 18.5, 63.1), positive regulation of cell population proliferation (29.8%, 95% CI: 18.5, 60.1), and positive regulation of cellular process (28.5%, 95% CI: 15.9, 61.6). The key proteins involved in these three processes primarily include IL15, FOLR2, REG3A, SIRT2, FABP3, and IL6 (Figure 6B). In sensitivity analyses, when proteins with suppression effects were reintroduced, only eight KEGG pathways remained significant in mediation analyses, with mediation proportions ranging from 5.7% to 12.2% (Figure S6). Similarly, only 98 GO Biological Processes remained significant mediators, with mediation proportions ranging from 3.0% to 21.2% (Figure S7).

## 3 Discussion

In this study, we included 288,180 participants to investigate the association between ambient benzene exposure and the risk of brain disorders, as well as the underlying biological mechanisms. We found that benzene exposure was significantly associated with seven major brain disorders, including anxiety disorders, dementia, epilepsy, MDD, migraine, SD, and SSD. Higher genetic risk appeared to confer greater susceptibility to benzene exposure for epilepsy, migraine, SD, and SSD, whereas this pattern was less evident for the other outcomes. When the influence of suppressive proteins was minimized, pathway‐level mediation analyses identified 230 proteins linking benzene exposure to brain disorders through 40 KEGG pathways and 253 GO Biological Processes, including the PI3K‐Akt signaling pathway, pathways in cancer, the MAPK signaling pathway, regulation of cell population proliferation, positive regulation of cell population proliferation, and positive regulation of cellular process. Reintroducing proteins with suppressive effects markedly attenuated pathway‐level mediation signals, suggesting that benzene‐related proteomic changes may include counteracting components that reduce net pathway effects. Our findings provide a foundation for future studies to clarify causality and underlying biology and highlight the importance of strengthening environmental regulation and developing precision prevention strategies.

In our study, ambient benzene exposure was significantly associated with seven major brain disorders, but not with PD. These results are consistent with previous epidemiological evidence. For example, a nested case‐control analysis from the UK showed that benzene exposure was associated with higher risks of several neuropsychiatric conditions, including mood disorders, anxiety disorders, substance use disorders, and SD [24]. We further conducted time‐dependent Cox regression and lag analyses, and the results were broadly consistent with those of the primary analyses, supporting the robustness of our findings. However, because benzene exposure may vary over time, baseline exposure may not fully capture long‐term exposure patterns during follow‐up. Therefore, potential misclassification of exposure and lagged effects cannot be completely excluded and should be considered when interpreting the results. We also found that individuals with higher genetic risk appeared to be more susceptible to the effects of benzene exposure on epilepsy, migraine, SD, and SSD. Previous studies have likewise shown that individuals with greater genetic vulnerability are more susceptible to the adverse neurological effects of air pollution, including PM_2.5_ [25, 26]. Our results are also consistent with those of previous studies, which also failed to detect statistically significant interactions between pollutant exposure and genetic risk [25, 26]. This pattern suggests that pollutant‐related neurotoxicity and genetic susceptibility may operate primarily through independent biological pathways, or that current analytical approaches may lack the power to detect subtle gene–environment interactions.

The exposure‐response pattern observed for PD warrants cautious interpretation. Rather than showing a monotonic increase in risk, the association followed an inverted V‐shaped pattern, with attenuation of the effect estimate in the highest exposure category. This pattern may reflect the long prodromal phase of PD and delays in clinical diagnosis, both of which can obscure associations between exposure and disease within a finite follow‐up period. It may also arise from sparse case numbers, statistical instability, or greater misclassification of exposure at the upper end of the exposure distribution [27]. In addition, benzene may not share the same pathogenic profile as pollutants more consistently implicated in PD, such as PM_2.5_ and NO_2_ [28, 29]. Although some studies have reported positive associations between air pollution and PD, the broader literature remains mixed, and epidemiological evidence specific to benzene is still sparse. Therefore, the attenuation observed at the highest exposure level should not be interpreted as evidence against a role for benzene in PD development; rather, it suggests that the association is complex and should be interpreted with caution.

Pathway‐level mediation analyses indicated that proteins associated with ambient benzene exposure were predominantly enriched in pathways involved in cell proliferation and apoptosis, intracellular signal transduction, and immune and inflammatory responses, consistent with existing toxicological evidence [30, 31, 32, 33, 34]. GO biological process analyses further supported and refined the KEGG findings, suggesting that benzene exposure may influence brain disorder risk through multiple interconnected potential biological pathways. Processes related to cellular homeostasis and programmed cell death were among the most strongly enriched GO terms, indicating that benzene‐associated alterations in protein profiles may enhance cellular stress responses while disrupting normal cellular renewal and repair. This pattern was consistent with the enrichment of canonical pathways such as PI3K‐Akt and MAPK. Previous studies have shown that the PI3K‐Akt pathway plays a central role in benzene‐induced hematopoietic toxicity and apoptosis [30, 34], supporting the relevance of dysregulated regulation of cell survival and death as a key potential mechanism underlying benzene‐related neurotoxicity.

Several GO terms implicated the regulation of intracellular signal transduction, including protein tyrosine phosphorylation, further supporting the notion that benzene exposure perturbs core kinase signaling networks and leads to broad downstream effects. Immune and inflammatory processes were also prominently enriched, including cytokine‐mediated signaling pathways and positive regulation of inflammatory responses, in line with the enrichment of TNF, NF‐κB, and JAK‐STAT signaling pathways. Prior studies have reported dose‐dependent transcriptomic alterations in cytokine‐related pathways following benzene exposure [30], as well as activation of the Aim2/Casp1 inflammasome and induction of pyroptosis by benzene metabolites [32]. Taken together, these features provide a rigorous conceptual and methodological basis for characterizing the potential biological pathways through which benzene exposure may contribute to brain disorders.

In the single‐protein mediation analysis, we identified 162 proteins with suppression effects on the association between benzene exposure and brain disorders. When these proteins were reintroduced in sensitivity analyses, the number of significant KEGG pathways and GO Biological Processes was markedly reduced, and the estimated mediation proportions were substantially attenuated. These findings suggest that benzene‐related proteomic changes are not uniformly aligned with the overall benzene‐brain disorder association. Instead, some proteins may change in the opposite direction, offsetting part of the positive mediation signal, attenuating the net pathway‐level mediated effect, and potentially obscuring biologically meaningful associations, thereby complicating the identification and interpretation of key mediating mechanisms [35]. Enrichment analyses showed that these suppression‐effect proteins were broadly distributed across immune‐related cell populations spanning both myeloid and lymphoid lineages, including activated T cells, CD4+ T cells, regulatory T cells, natural killer cells, monocytes, macrophages, and microglia. GTEx analysis also supported distinct tissue‐expression contexts, with enrichment in whole blood‐, lung‐, and vascular‐related signatures. Taken together, these findings suggest that benzene exposure may disrupt hematopoietic and immune homeostasis while eliciting complex circulating proteomic responses, some of which may reflect immune‐regulatory, feedback‐inhibitory, or compensatory processes rather than solely risk‐promoting signals [36, 37].

This study has several strengths. To our knowledge, it is among the few to examine the potential mechanistic links between benzene exposure and brain disorders. First, we generated PRS for eight major brain disorders and systematically quantified gene–environment interactions. This allowed us to delineate how inherited susceptibility shapes individual vulnerability to benzene‐associated neurological risks. Second, we introduced a multidimensional mediation framework capable of resolving both protein‐level effects and the aggregate influence of biological pathways. This framework confers two major advantages: it reduces bias arising from proteins with masking or suppressor effects, and it pinpoints the dominant pathways showing stronger statistical mediation signals across the observed exposure‐disease association. These features, when considered together, provide a rigorous conceptual and methodological basis for characterizing the potential biological pathways underlying benzene exposure's contribution to brain disorders [38].

However, several limitations should be acknowledged. First, because individual‐level exposure data were unavailable, benzene exposure was estimated using a 1 × 1 km ambient concentration surface rather than personal measurements. This approach likely introduced exposure misclassification, particularly because it failed to capture within‐grid spatial variability, individual time‐activity patterns, commuting, and indoor exposure. Because such misclassification is likely to be largely non‐differential with respect to disease status, it would most likely bias the observed associations toward the null, although the extent of this bias could not be quantified in the present study. Second, although we adjusted for a broad range of covariates, residual confounding cannot be excluded. In particular, our dataset did not capture several potential sources of indoor benzene exposure and related air pollution, such as household solvent use, environmental tobacco smoke, cooking fuels, and other domestic combustion sources. Third, our proteomic measurements were derived from plasma and may not fully reflect molecular changes in the brain. This is an important limitation for biological interpretation, as circulating proteins may primarily capture peripheral responses rather than brain‐specific processes. Accordingly, the mediation findings should not be interpreted as evidence of causal mechanisms, but rather as identifying circulating protein signatures and potential biological pathways that may be involved in the association between benzene exposure and brain disorders. Fourth, our pathway‐level mediation analysis did not account for the hierarchical or sequential regulatory structure of proteins within biological pathways. Because many pathways rely on ordered activation cascades and feedback relationships, the estimated joint mediation effects may not fully capture the complexity of pathway regulation. Finally, the UKB cohort consists mainly of relatively healthy urban residents, which may limit the generalizability of our findings. In many rural settings, reliance on solid fuels and limited regulation of open burning may lead to benzene exposures that differ from those observed in urban environments, underscoring the need for future studies in more diverse populations.

In conclusion, ambient benzene exposure was associated with an increased risk of multiple brain disorders, with stronger associations observed among individuals with higher genetic susceptibility. After excluding 162 proteins showing suppression effects, which were associated with heterogeneous immune‐related contexts rather than a single dominant lineage, pathway‐level mediation analyses suggested that these associations were primarily related to coordinated protein‐mediated processes involving regulation of cell survival and death, intracellular signaling, and immune‐inflammatory responses, with PI3K‐Akt and MAPK signaling emerging as prominent nodes. These findings suggest that even relatively low‐level ambient benzene exposure may adversely affect brain health through multiple potential biological pathways. Given that benzene exposure may be even higher in some rural settings, particularly where solid fuel combustion and open burning are common, future studies should extend this work to more diverse populations and exposure environments.

## 4 Materials and Methods

### 4.1 Study Design

We employed Cox proportional hazards regression models to evaluate the associations between benzene exposure and eight major brain disorders, including anxiety disorders, dementia, epilepsy, MDD, migraine, PD, SD, and SSD. PRS were calculated to explore the interaction between benzene exposure and genetic susceptibility in relation to the risk of these brain disorders. Using the Olink proteomics platform, we developed a structured mediation framework to quantify the extent to which individual proteins mediate the association between benzene exposure and brain disorders. In addition, we grouped proteins by their annotated biological pathways to estimate pathway‐level mediation effects.

### 4.2 Study Population and Brain Disorders Diagnosis

The study population was derived from the UKB cohort, comprising 502,164 individuals enrolled between 2006 and 2010. Follow‐up continued from baseline until the earliest occurrence of incident brain disorders, death, withdrawal from the study, or the censoring date (1 September 2023). Anxiety disorders, dementia, epilepsy, MDD, migraine, PD, SD, and SSD were ascertained from the UK Biobank Health Outcomes dataset using the relevant International Classification of Diseases codes. Case identification drew on linked primary care records, hospital admissions, death registries, and algorithmically derived endpoints. The diagnosis codes are detailed in Table S1. Eligible participants were restricted to individuals residing in urban areas, as defined by field ID 20118, and to those with complete outcome data. We excluded individuals with a pre‐existing diagnosis of any of the eight brain disorders at baseline, those with extreme genotype heterozygosity or high missingness, and those who were third‐degree or more closely related to another participant. In total, 288,180 individuals aged 40–69 years who consented to participate were included in the analysis, of whom 37,273 had available proteomic data for mediation analyses. Missing values were imputed using multiple imputation implemented by chained equations [39]. UKB participation is entirely voluntary and not financially incentivized. The study received ethical approval from the North West Multi‐centre Research Ethics Committee, and all analyses were performed under UKB application 199688. The study report complied with the STROBE guidelines for cohort studies.

### 4.3 Assessment of Benzene Exposure

Benzene exposure was estimated using annual 1 km × 1 km benzene concentration surfaces derived from the UK Pollution Climate Mapping (PCM) model [40, 41, 42]. The PCM model estimates annual mean background benzene concentrations by combining contributions from rural background, combustion point sources, fugitive and process point sources, emissions trading scheme (ETS) point sources, and local area sources, while concentrations near major urban roads are derived by adding a roadside increment calculated using the PCM Roads Kernel Model (PCM‐RKM) [40, 43]. In the 2023 technical assessment, the benzene background model was calibrated using measurements from one site in the national automatic hydrocarbon network and 21 non‐automatic hydrocarbon sites, and the roadside model was calibrated using measurements from nine non‐automatic hydrocarbon sites. The model is driven by emissions from the UK National Atmospheric Emissions Inventory (NAEI) and meteorological data from the Weather Research and Forecasting (WRF) model [41]. For the main Cox proportional hazards models, benzene exposure was defined as the average of annual mean benzene concentrations over 2003–2010 before the study baseline and linked to each participant's residential address. Concentrations were further standardized using the IQR = 0.24 µg/m^3^ and categorized into three exposure levels based on the first and third quartiles: low (≤0.58 µg/m^3^), moderate (0.58–0.82 µg/m^3^), and high (>0.82 µg/m^3^). For time‐varying analyses, annual benzene exposure was updated during follow‐up, with lag 0, lag 1, lag 2, and lag 3 representing exposure in the same year and 1, 2, and 3 years before the year at risk, respectively. The corresponding IQRs were 0.19 µg/m^3^ for lag 0, 1, and 2, and 0.21 µg/m^3^ for lag 3.

### 4.4 PRS of Brain Disorders

PRS were constructed using summary statistics from large‐scale genome‐wide association studies (GWAS). Summary statistics for MDD and anxiety disorders were obtained from the Psychiatric Genomics Consortium (PGC) [44, 45]. To comply with data‐access restrictions and avoid potential sample overlap, individuals from the UKB and 23andMe cohorts were excluded from these datasets. Summary statistics for dementia, epilepsy, migraine, SD, and SSD were derived from the FinnGen R11 release. Because FinnGen GWAS results were aligned to the GRCh38 reference genome, we lifted them over to GRCh37 to align with the UK Biobank coordinate system. Only SNPs with a minor allele frequency >0.01 and an imputation quality (INFO) score >0.80 were retained for PRS derivation. PRS were generated using the clumping‐and‐thresholding (C+T) approach implemented in PRSice‐2 [46]. Linkage disequilibrium (LD) clumping was performed using UK Biobank genotypes as the reference, with an *r* ^2^ threshold of 0.1 and a 250‐kb window. Scores were computed across a sequence of *p*‐value thresholds ranging from 5 × 10^−8^ to 1.0, and PRSice‐2 automatically selected the optimal threshold based on the highest PRS‐R^2^. SNP weights were derived from GWAS effect sizes (*β*) using the *β*‐scoring mode. For binary phenotypes, associations between PRS and disease outcomes were evaluated using logistic regression models adjusted for age, sex, and the first 10 principal components. Empirical significance was assessed using 10,000 permutation iterations. Detailed information on the GWAS summary statistics for each disease, including total sample size, numbers of cases and controls, and the number of SNPs retained after quality control, is provided in Table S2. A detailed summary of PRS performance across *p*‐value thresholds and the optimal models for each brain disorder phenotype is shown in Tables S3 and S4. In addition, the PRS for PD were obtained directly from the UK Biobank (field ID 26260); the computational procedures underlying these PRS have been described in detail previously [47, 48]. Standardized PRS distributions for all disease phenotypes are presented in Figure S1.

### 4.5 Proteomic Platforms

Proteomic profiling was conducted using two high‐throughput platforms, Olink Explore 3072 and SomaScan v4. The Olink Explore platform employs proximity extension assays, in which paired antibodies, each linked to complementary oligonucleotides, bind the target protein and generate a unique DNA barcode that is quantified by next‐generation sequencing, producing normalized NPX values on a log_2_ scale [49, 50]. The SomaScan platform uses aptamer‐based SOMAmer reagents that bind protein targets and are quantified via DNA microarrays; both non‐normalized and SMP‐normalized datasets were analyzed [51]. Across proteins measured by both platforms, dilution group classifications showed strong concordance [52]. All assays underwent standard platform‐specific quality control and normalization, and the resulting measurements represent assay‐derived protein levels rather than absolute concentrations [53, 54].

### 4.6 Covariates

All covariates were assessed at baseline, including demographic characteristics, lifestyle factors, socioeconomic status, and anthropometric measures. Based on prior evidence, the following variables were included: age, sex, qualifications, body mass index (BMI), Charlson Comorbidity Index (CCI) [55], occupational benzene exposure [11], alcohol consumption [56, 57], smoking history, moderate sleep, moderate physical activity (weekly MET‐minutes of moderate activity), healthy diet score [58], proximity to major roads (inverse distance to the nearest major road), and noise pollution (average 24‐h noise exposure level). Detailed definitions of these covariates are provided in Table S5. The algorithm used to calculate the CCI is detailed in Table S6.

### 4.7 Statistical Analyses

Cox proportional hazards models were used to examine the associations between benzene exposure and brain disorders. The proportional hazards assumption was verified for all models using scaled Schoenfeld residuals. Three modeling strategies were applied to address potential confounding: (1) an unadjusted model (Model 1); (2) a partially adjusted model controlling for age, sex, qualifications, BMI, and the CCI (Model 2); and (3) a fully adjusted model that further included occupational benzene exposure, alcohol consumption, smoking history, moderate sleep, moderate physical activity, healthy diet score, proximity to major roads, and noise pollution (Model 3). Effect estimates were reported as HRs with corresponding 95% CIs, using the lowest exposure category as the reference, and as per‐IQR increases in benzene concentration. To assess the robustness of the findings, we additionally adjusted for PM_2.5_, NO_2_, and NOx concentrations in separate sensitivity analyses. We also performed sensitivity analyses using time‐dependent Cox regression models to account for temporal variation in benzene exposure. Annual benzene concentrations were incorporated as time‐varying exposures, and lag effects were examined by relating each risk set to benzene exposure in the same year (lag 0), as well as to exposure in the previous 1, 2, and 3 years. Exposure‐response relationships were explored using natural cubic spline regression, adjusting for all Model 3 covariates, with knots placed at the 25^th^, 50^th^, and 75^th^ percentiles of the exposure distribution.

We further investigated the joint and interactive effects of benzene exposure and genetic susceptibility on brain disorders. Participants were categorized into nine groups based on tertiles of benzene exposure and tertiles of the PRS for each brain disorder. Cox proportional hazards models were used to estimate risk across all exposure‐genetic risk combinations, with the group characterized by both low genetic risk and low benzene exposure serving as the reference. In addition, additive interactions between benzene exposure and genetic risk were assessed for each of the eight brain disorders. These analyses compared the joint high‐exposure/high‐genetic‐risk group with the joint low‐exposure/low‐genetic‐risk group, and three measures of additive interaction were reported: the relative excess risk due to interaction (RERI), the synergy index (SI), and the attributable proportion due to interaction (AP).

To systematically investigate the role of circulating proteins as putative mediators in the relationship between benzene exposure and brain disorders, we developed a multidimensional mediation analysis framework. Candidate proteins were first selected based on their associations with both benzene exposure and brain disorders: multiple linear regression models were used to evaluate the association between benzene exposure and circulating proteins, and Cox proportional hazards models were used to assess the association between these proteins and brain disorder risk. Proteins significantly associated with both exposure and outcome were retained for subsequent mediation analyses.

In the first stage, we conducted single‐protein mediation analyses for all candidate proteins. Functional enrichment analysis was performed on all candidate proteins using Enrichr (accessed on 14 December 2025). This included KEGG and GO categories (biological process, molecular function, and cellular component) as well as Reactome pathways, to identify the biological processes and pathways involved [59]. We then characterized the tissue and cell‐type distributions of both suppression‐effect proteins and positively mediating proteins using CellMarker and GTEx to delineate their biological localization. In the second stage, all proteins mapped to the same biological pathway were jointly included in pathway‐level mediation models to quantify their combined mediating effects. Mediation analyses were conducted using the regression‐based approach (“rb” model) implemented in the *CMAverse* package [60]. Benzene exposure was specified as the exposure, proteins as mediators, and brain disorders as time‐to‐event outcomes modelled using Cox proportional hazards models. Mediator models were fitted with linear regression and adjusted for all covariates. Mediators were mean‐centered, and no interaction term between exposure and mediator was included. Statistical inference was based on 1,000 nonparametric bootstrap resamples with percentile‐based confidence intervals. Because proteins with suppression effects may represent biologically distinct or directionally inconsistent mediation signals, the primary pathway‐level analyses focused on proteins with concordant mediation effects. To assess the influence of suppression‐effect proteins on pathway‐level estimates, we additionally performed sensitivity analyses in which these proteins were reintroduced into the corresponding pathway‐level mediation models.

## Author Contributions

J.G., S.H., and J.L. had full access to all data and take responsibility for the integrity of the data and the accuracy of the analyses. J.G., S.H., and J.L. conceived and designed the study. Data were acquired, analyzed, and interpreted by all authors. Statistical analyses were done by J.G., X.Z., and S.H. The manuscript was drafted by J.G., P.K., C.L.Z.V., F.S., S.H., and J.L., and critically revised by Y.F., Y.W., X.L., Z.W., F.S., and J.L. All authors reviewed and approved the final version.

## Funding

This work was supported by the National Key Research and Development Program of China (Grant No. 2023YFC3709302), the National Natural Science Foundation of China (Grant No.42307133), the Capital's Funds for Health Improvement and Research (Grant No. CFH 2026‐2G‐4405), the Young Elite Scientists Sponsorship Program by CAST (Grant No. 2023QNRC001), and the Special Fund of the State Key Joint Laboratory of Environmental Simulation and Pollution Control (Grant No. 23K02ESPCP). This research has been conducted using the UK Biobank Resource under Application Number [199688]. We thank the UK Biobank participants and coordinators for their invaluable contributions. The High‐performance Computing Platform of Peking University also supported this study.

## Conflicts of Interest

The authors declare no conflict of interest.

## Supporting information




**Supporting File**: advs75874‐sup‐0001‐SuppMat.docx.

## Data Availability

The data used in the present study are available from UKB with restrictions applied. Data were used under license and are therefore not publicly available. Access can be obtained by applying to the UK Biobank through the standard protocol (https://www.ukbiobank.ac.uk/register‐apply/).
