# Supplementary material for: Long‐Term Ambient Benzene Exposure and Brain Disorders Among Urban Adults: Effect Modification by Genetic Susceptibility and Potential Mediation by Plasma Proteins
Source: Adv Sci (Weinh). 2026 May 29;13(47):e75874. doi: 10.1002/advs.75874 (PMC13336048; doi:10.1002/advs.75874)
Supplement: Supplementary file 1 [file ADVS-13-e75874-s001.docx]

**Supplementary Information**

**Long-term ambient benzene exposure and brain disorders among urban adults: effect modification by genetic susceptibility and potential mediation by plasma proteins**

Jianhui Guo^1, #^, Xia Zhong^1, #^, Petros Koutrakis^2^, Carolina L. Zilli Vieira^2^, Feifei Si^3^, Yi Fan^4^, Yaqi Wang^1^, Xinyao Lian^1^, Zhaokun Wang^4^, Shaodan Huang^4,5^, Jing Li^1, *^

**Affiliations:**

^1^ Institute of Child and Adolescent Health, School of Public Health, Peking University; National Health Commission Key Laboratory of Reproductive Health, Beijing 100191, China.

^2^ Department of Environmental Health, Harvard T.H. Chan School of Public Health, Boston 02115, USA.

^3^ Peking University Sixth Hospital/Institute of Mental Health; NHC Key Laboratory of Mental Health (Peking University), National Clinical Research Center for Mental Disorders (Peking University Sixth Hospital), Beijing 100191, China.

^4^ Department of Occupational and Environmental Health Sciences, School of Public Health, Peking University, Beijing 100191, China.

^5^ Key Laboratory of Epidemiology of Major Diseases (Peking University), Ministry of Education, Beijing 100191, China

**^#^ Contributed equally to this work.**

*** Corresponding Authors**

**Jing Li** - Institute of Child and Adolescent Health, School of Public Health, Peking University; National Health Commission Key Laboratory of Reproductive Health, Beijing 100191, China; ORCID: 0000-0001-7682-4311; Email: jing.li@hsc.pku.edu.cn (Jing Li).

**Table of Contents**

[Supplementary Figure 1. Distribution of standardised polygenic risk scores for major brain disorders among cases and controls.	4](#_Toc227681980)

[Supplementary Figure 2. Annual ambient benzene concentrations in the UK from 2003 to 2010.	5](#_Toc227681981)

[Supplementary Figure 3. Subgroup analyses of the association between benzene exposure and incident brain disorders.	6](#_Toc227681982)

[Supplementary Figure 4. Sensitivity analyses of the association between benzene exposure and incident brain disorders.	7](#_Toc227681983)

[Supplementary Figure 5. Bioinformatic analysis of the 392 identified proteins.	8](#_Toc227681984)

[Supplementary Figure 6. Comparison of pathway-level mediation effects for KEGG pathways derived from positively mediating proteins versus all detected proteins.	9](#_Toc227681985)

[Supplementary Figure 7. Comparison of pathway-level mediation effects for GO biological process pathways derived from positively mediating proteins versus all detected proteins.	10](#_Toc227681986)

[Supplementary Table 1. International Classification of Diseases (ICD-10) Codes for brain disorders.	12](#_Toc227681987)

[Supplementary Table 2. Summary of GWAS sources and characteristics for brain disorders.	13](#_Toc227681988)

[Supplementary Table 3. PRSice-2 summary of polygenic risk score performance across P-value thresholds.	14](#_Toc227681989)

[Supplementary Table 4. Optimal polygenic risk score models for brain disorder phenotypes.	15](#_Toc227681990)

[Supplementary Table 5. Definitions and coding of covariates used in the analyses.	16](#_Toc227681991)

[Supplementary Table 6. Definitions of the Charlson Comorbidity Index.	18](#_Toc227681992)

[Supplementary Table 7. Baseline characteristics of the studied population.	19](#_Toc227681993)

[Supplementary Table 8. Association between ambient benzene exposure and brain disorders.	21](#_Toc227681994)

[Supplementary Table 9. Sensitivity analysis of the associations between ambient benzene exposure and brain disorders using time-dependent Cox regression models.	22](#_Toc227681995)

[Supplementary Table 10. Risk of incident brain disorders according to categories of benzene exposure within each genetic risk stratum.	23](#_Toc227681996)

[Supplementary Table 11. Additive interaction between benzene exposure and genetic risk on brain disorders.	25](#_Toc227681997)

[Supplementary Table 12. Mediation analysis results for proteins in the association between benzene exposure and brain disorders.	27](#_Toc227681998)

[Supplementary Table 13. The results of CellMarker and GTEx.	28](#_Toc227681999)

[Supplementary Table 14. Pathway-level mediation analysis of KEGG pathways based on positively mediating proteins.	30](#_Toc227682000)

[Supplementary Table 15. Pathway-level mediation analysis of GO biological process pathways based on positively mediating proteins.	32](#_Toc227682001)

# **Supplementary Figure 1. Distribution of standardised polygenic risk scores for major brain disorders among cases and controls.**

MDD, major depressive disorder; PD, Parkinson’s disease; SD, Sleep disorders; SSD, schizophrenia spectrum disorders.

# **Supplementary Figure 2. Annual ambient benzene concentrations in the UK from 2003 to 2010.**

# **Supplementary Figure 3. Subgroup analyses of the association between benzene exposure and incident brain disorders.**

Hazard ratios with 95% confidence intervals are presented across strata of sex, age, educational attainment, alcohol consumption, and smoking history to evaluate potential effect modification. All analyses were adjusted for age, sex, qualifications, body mass index (BMI), Charlson Comorbidity Index (CCI), occupational benzene exposure, alcohol consumption, smoking history, moderate sleep, moderate physical activity, healthy diet score, proximity to major roads, and noise pollution. MDD, major depressive disorder; PD, Parkinson’s disease; SD, Sleep disorders; SSD, schizophrenia spectrum disorders.

# **Supplementary Figure 4. Sensitivity analyses of the association between benzene exposure and incident brain disorders.**

All models were adjusted for age, sex, education, body mass index, Charlson Comorbidity Index (CCI), occupational benzene exposure, alcohol consumption, smoking history, sleep duration, moderate physical activity, healthy diet score, proximity to major roads, and noise pollution, with PM_2.5_ (Model 1), NO_2_ (Model 2), and NOx (Model 3) each further added separately for sensitivity analyses. MDD, major depressive disorder; PD, Parkinson’s disease; SD, Sleep disorders; SSD, schizophrenia spectrum disorders.

# **Supplementary Figure 5. Bioinformatic analysis of the 392 identified proteins.**

# **Supplementary Figure 6. Comparison of pathway-level mediation effects for KEGG pathways derived from positively mediating proteins versus all detected proteins.**

Pathway-level mediation proportions were estimated by jointly modeling all positively mediating proteins (Model 1) and all detected proteins (Model 2) within each pathway. All analyses were adjusted for age, sex, qualifications, BMI, Charlson Comorbidity Index (CCI), occupational benzene exposure, alcohol consumption, smoking history, moderate sleep, moderate physical activity, healthy diet score, proximity to major roads, and noise pollution. Statistical significance was assessed using two-sided Z-tests.

# **Supplementary Figure 7. Comparison of pathway-level mediation effects for GO biological process pathways derived from positively mediating proteins versus all detected proteins.**

Pathway-level mediation proportions were estimated by jointly modeling all positively mediating proteins (Model 1) and all detected proteins (Model 2) within each pathway. All analyses were adjusted for age, sex, qualifications, BMI, Charlson Comorbidity Index (CCI), occupational benzene exposure, alcohol consumption, smoking history, moderate sleep, moderate physical activity, healthy diet score, proximity to major roads, and noise pollution. Statistical significance was assessed using two-sided Z-tests.

# **Supplementary Table 1. International Classification of Diseases (ICD-10) Codes for brain disorders.**

| **Diseases** | **ICD-10 Codes** | **Field ID** |
| --- | --- | --- |
| Anxiety | F40 and F41 | 130904 and 130906 |
| Dementia | F00, F01, F02, F03, and G30 | 130836, 130838, 130840, 130842, and 131036 |
| Epilepsy | G40 and G41 | 131048 and 131050 |
| MDD | F32 and F33 | 130894 and 130896 |
| Migraine | G43 | 131052 |
| PD | G20 | 131022 |
| SD | F51and G47 | 130920 and 131060 |
| SSD | F20, F21, F22, F23, F24, F25, F28, and F29 | 130874, 130876, 130878, 130880, 130882, 130884, 130886, and 130888 |

MDD, major depressive disorder; PD, Parkinson’s disease; SD, Sleep disorders; SSD, schizophrenia spectrum disorders.

# **Supplementary Table 2. Summary of GWAS sources and characteristics for brain disorders.**

| **Diseases** | **Source** | **Case** | **Control** | **SNP number** |
| --- | --- | --- | --- | --- |
| Anxiety | Psychiatric Genomics Consortium | 2,147 | 7,760 | 10,151,624 |
| Dementia | FinnGen R11 | 20,667 | 428,053 | 20,444,616 |
| Epilepsy | FinnGen R11 | 14,089 | 341,884 | 20,443,104 |
| MDD | Psychiatric Genomics Consortium | 357,636 | 1,281,936 | 7139,872 |
| Migraine | UK Biobank | - | - | - |
| PD | UK Biobank | - | - | - |
| SD | FinnGen R11 | 57,251 | 391,891 | 20,444,636 |
| SSD | FinnGen R11 | 14529 | 330460 | 21,304,465 |

MDD, major depressive disorder; PD, Parkinson’s disease; SD, Sleep disorders; SSD, schizophrenia spectrum disorders.

# **Supplementary Table 3. PRSice-2 summary of polygenic risk score performance across P-value thresholds.**

| **Phenotype** | **Set** | **Threshold** | **R^2^** | **P** | **Coefficient** | **Standard Error** | **Number of SNPs** |
| --- | --- | --- | --- | --- | --- | --- | --- |
| Anxiety | Base | 0.001 | 1.75×10^-04^ | 1.01×10^-04^ | 22.5 | 5.8 | 935 |
| Anxiety | Base | 0.050 | 3.37×10^-04^ | 7.26×10^-08^ | 190.7 | 35.4 | 23744 |
| Anxiety | Base | 0.100 | 3.90×10^-04^ | 6.69×10^-09^ | 286.1 | 49.3 | 41296 |
| Dementia | Base | 0.001 | 1.02×10^-02^ | 3.76×10^-106^ | 781.2 | 35.7 | 2627 |
| Dementia | Base | 0.050 | 4.53×10^-03^ | 8.43×10^-48^ | 3522.1 | 242.5 | 45775 |
| Dementia | Base | 0.100 | 3.60×10^-03^ | 2.14×10^-38^ | 4394.2 | 339.1 | 76473 |
| Epilepsy | Base | 0.001 | 1.98×10^-05^ | 5.00×10^-01^ | 36.3 | 53.9 | 1666 |
| Epilepsy | Base | 0.050 | 1.48×10^-04^ | 6.52×10^-02^ | 643.2 | 348.8 | 41766 |
| Epilepsy | Base | 0.100 | 1.82×10^-04^ | 4.09×10^-02^ | 1000.9 | 489.6 | 71843 |
| MDD | Base | 0.001 | 6.52×10^-03^ | 3.68×10^-123^ | 2954.6 | 125.2 | 7212 |
| MDD | Base | 0.050 | 8.41×10^-03^ | 9.17×10^-159^ | 14381.3 | 535.7 | 66229 |
| MDD | Base | 0.100 | 8.23×10^-03^ | 2.37×10^-155^ | 19209.5 | 723.4 | 102172 |
| Migraine | Base | 0.001 | 8.85×10^-04^ | 3.09×10^-08^ | 367.4 | 66.4 | 2829 |
| Migraine | Base | 0.050 | 2.00×10^-03^ | 8.69×10^-17^ | 2892.7 | 347.6 | 48668 |
| Migraine | Base | 0.100 | 1.99×10^-03^ | 9.57×10^-17^ | 3969.5 | 477.7 | 79808 |
| SD | Base | 0.001 | 1.84×10^-03^ | 7.48×10^-23^ | 844.2 | 85.8 | 3779 |
| SD | Base | 0.050 | 3.50×10^-03^ | 7.84×10^-42^ | 5535.3 | 408.5 | 52944 |
| SD | Base | 0.100 | 3.67×10^-03^ | 8.05×10^-44^ | 7697.2 | 554.4 | 84715 |
| SSD | Base | 0.001 | 2.78×10^-04^ | 1.37×10^-01^ | 115.6 | 77.7 | 2076 |
| SSD | Base | 0.050 | 1.40×10^-03^ | 8.19×10^-04^ | 1672.4 | 499.8 | 44631 |
| SSD | Base | 0.100 | 1.23×10^-03^ | 1.71×10^-03^ | 2192.6 | 699.1 | 75535 |

MDD, major depressive disorder; PD, Parkinson’s disease; SD, Sleep disorders; SSD, schizophrenia spectrum disorders.

# **Supplementary Table 4. Optimal polygenic risk score models for brain disorder phenotypes.**

| **Phenotype** | **Set** | **Threshold** | **PRS R^2^** | **Full R^2^** | **Null R^2^** | **Coefficient** | **Standard error** | **P** | **Number of SNPs** | **Empirical-P** |
| --- | --- | --- | --- | --- | --- | --- | --- | --- | --- | --- |
| Anxiety | Base | 0.100 | 3.94×10^-04^ | 1.08×10^-02^ | 1.04×10^-02^ | 286.1 | 49.3 | 6.69×10^-09^ | 41296 | 1.00×10^-04^ |
| Dementia | Base | 0.001 | 1.19×10^-02^ | 1.51×10^-01^ | 1.41×10^-01^ | 781.2 | 35.7 | 3.76×10^-106^ | 2627 | 1.00×10^-04^ |
| Epilepsy | Base | 0.100 | 1.84×10^-04^ | 1.42×10^-02^ | 1.40×10^-02^ | 1000.9 | 489.6 | 4.09×10^-02^ | 71843 | 8.99×10^-02^ |
| MDD | Base | 0.050 | 8.45×10^-03^ | 1.27×10^-02^ | 4.28×10^-03^ | 14381.3 | 535.7 | 9.17×10^-159^ | 66229 | 1.00×10^-04^ |
| Migraine | Base | 0.050 | 2.03×10^-03^ | 1.60×10^-02^ | 1.40×10^-02^ | 2892.7 | 347.6 | 8.69×10^-17^ | 48668 | 1.00×10^-04^ |
| SD | Base | 0.100 | 3.72×10^-03^ | 1.63×10^-02^ | 1.26×10^-02^ | 7697.2 | 554.4 | 8.05×10^-44^ | 84715 | 1.00×10^-04^ |
| SSD | Base | 0.050 | 1.42×10^-03^ | 1.23×10^-02^ | 1.09×10^-02^ | 1672.4 | 499.8 | 8.19×10^-04^ | 44631 | 2.30×10^-03^ |

This table presents the optimal PRS models identified by PRSice-2 for each neuropsychiatric phenotype across various P-value thresholds. For each trait, the threshold that maximised predictive performance is shown, along with model fit statistics and SNP counts. MDD, major depressive disorder; PD, Parkinson’s disease; SD, Sleep disorders; SSD, schizophrenia spectrum disorders.

# **Supplementary Table 5. Definitions and coding of covariates used in the analyses.**

| **Field ID** | **Variable name** | **Original code** | **New code** | **Labels** |
| --- | --- | --- | --- | --- |
| 21022 | Age at recruitment |  |  |  |
| 31 | Sex | Female | 0 | Female |
|  |  | Male | 1 | Male |
| 6138 | Qualifications | O levels/GCSEs or equivalent | 1 | Lower education |
|  |  | College or University degree | 0 | Higher education |
|  |  | Other professional qualifications eg: nursing, teaching | 0 | Higher education |
|  |  | NVQ or HND or HNC or equivalent | 1 | Lower education |
|  |  | A levels/AS levels or equivalent | 1 | Lower education |
|  |  | CSEs or equivalent | 1 | Lower education |
|  |  | None of the above | 1 | Lower education |
| 22038 | Moderate physical activity |  |  |  |
| 20160 | Ever smoked | Yes | 1 | Yes |
|  |  | No | 0 | No |
| 1160 | Moderate sleep | <7 | 1 | Improper sleep duration |
|  |  | >9 | 1 | Improper sleep duration |
|  |  | 7~9 | 0 | Moderate sleep duration |
| - | Healthy diet score ^δ^ |  |  |  |
| - | Alcohol consumption ^ζ^ |  | 0 | Drink moderately |
|  |  |  | 1 | Inappropriate drinking |
| 24012 | Inverse distance to the nearest major road |  |  |  |
| 24024 | Average 24-hour sound level of noise pollution |  |  |  |
| 23104 | Body mass index |  |  |  |
| 20118 | Home area population density - urban or rural | England/Wales - Urban - sparse | 1 |  |
|  |  | England/Wales - Town and Fringe - sparse | 0 |  |
|  |  | England/Wales - Village - sparse | 0 |  |
|  |  | England/Wales - Hamlet and Isolated dwelling - sparse | 0 |  |
|  |  | England/Wales - Urban - less sparse | 1 |  |
|  |  | England/Wales - Town and Fringe - less sparse | 0 |  |
|  |  | England/Wales - Village - less sparse | 0 |  |
|  |  | England/Wales - Hamlet and Isolated Dwelling - less sparse | 0 |  |
|  |  | Postcode not linkable | 0 |  |
|  |  | Scotland - Large Urban Area | 1 |  |
|  |  | Scotland - Other Urban Area | 1 |  |
|  |  | Scotland - Accessible Small Town | 0 |  |
|  |  | Scotland - Remote Small Town | 0 |  |
|  |  | Scotland - Very Remote Small Town | 0 |  |
|  |  | Scotland - Accessible Rural | 0 |  |
|  |  | Scotland - Remote Rural | 0 |  |
|  |  | Scotland - Very Remote Rural | 0 |  |

^δ^ Derived from UKB field codes 1289, 1299, 1309, 1319, 1329, 1339, 1438, 1448, 1458, 1468, 1369, 1379, 1389, and 1349.

^ζ^ Derived from UKB field codes 1568, 4407, 1578, 4418, 1588, 4429, 1598, 4440,1608, 4451, 5364, and 4462.

# **Supplementary Table 6. Definitions of the Charlson Comorbidity Index.**

| **Item** | **UKB data field** | **Score** |
| --- | --- | --- |
| Age <50 | 21022 | 0 |
| Age 50–59 | 21022 | 1 |
| Age 60–69 | 21022 | 2 |
| Age 70–79 | 21022 | 3 |
| Age ≥80 | 21022 | 4 |
| Myocardial infarction | Self-reported baseline non-cancer illness codes (20002) and ICD^-10^ codes before/at baseline (41270) | 1 |
| Congestive heart failure | Self-reported baseline non-cancer illness codes (20002) and ICD^-10^ codes before/at baseline (41270) | 1 |
| Peripheral vascular disease | Self-reported baseline non-cancer illness codes (20002) and ICD^-10^ codes before/at baseline (41270) | 1 |
| Cerebrovascular disease | Self-reported baseline non-cancer illness codes (20002) and ICD^-10^ codes before/at baseline (41270) | 1 |
| Hemiplegia or paraplegia | Self-reported baseline non-cancer illness codes (20002) and ICD^-10^ codes before/at baseline (41270) | 2 |
| Dementia | Self-reported baseline non-cancer illness codes (20002) and ICD^-10^ codes before/at baseline (41270) | 1 |
| Chronic pulmonary disease | Self-reported baseline non-cancer illness codes (20002) and ICD^-10^ codes before/at baseline (41270) | 1 |
| Rheumatologic disease | Self-reported baseline non-cancer illness codes (20002) and ICD^-10^ codes before/at baseline (41270) | 1 |
| Peptic ulcer disease | Self-reported baseline non-cancer illness codes (20002) and ICD^-10^ codes before/at baseline (41270) | 1 |
| Diabetes without chronic complications | Self-reported baseline non-cancer illness codes (20002) and ICD^-10^ codes before/at baseline (41270) | 1 |
| Diabetes with chronic complications | Self-reported baseline non-cancer illness codes (20002) and ICD^-10^ codes before/at baseline (41270) | 2 |
| Chronic kidney disease | Self-reported baseline non-cancer illness codes (20002) and ICD^-10^ codes before/at baseline (41270) | 2 |
| Malignancy | Self-reported cancer codes (20001) and ICD^-10^ codes before/at baseline (41270) | 2 |
| Metastatic solid tumor | Self-reported cancer codes (20001) and ICD^-10^ codes before/at baseline (41270) | 6 |
| Mild liver disease | Self-reported baseline non-cancer illness codes (20002) and ICD^-10^ codes before/at baseline (41270) | 1 |
| Moderate or severe liver disease | Self-reported baseline non-cancer illness codes (20002) and ICD^-10^ codes before/at baseline (41270) | 3 |
| AIDS/HIV | Self-reported baseline non-cancer illness codes (20002) and ICD^-10^ codes before/at baseline (41270) | 6 |

# **Supplementary Table 7. Baseline characteristics of the studied population.**

| **Variable** | **All** | **Anxiety** | | **Dementia** | | **Epilepsy** | | **MDD** | | **Migraine** | | **PD** | | **SD** | | **SSD** | |
| --- | --- | --- | --- | --- | --- | --- | --- | --- | --- | --- | --- | --- | --- | --- | --- | --- | --- |
|  |  | **Non-cases** | **Case** | **Non-cases** | **Case** | **Non-cases** | **Case** | **Non-cases** | **Case** | **Non-cases** | **Case** | **Non-cases** | **Case** | **Non-cases** | **Case** | **Non-cases** | **Case** |
| **Sample size [n (%)]** | 288180 (100.2) | 275851 (95.9) | 12329 (4.3) | 283283 (98.5) | 4897 (1.7) | 286177 (99.5) | 2003 (0.7) | 275861 (95.9) | 12319 (4.3) | 284802 (99.0) | 3378 (1.2) | 286225 (99.5) | 1955 (0.7) | 282262 (98.1) | 5918 (2.1) | 287624 (100.0) | 556 (0.2) |
| **Follow-up time [mean (SD)], years** | 14.0 (2.25) | 14.0 (2.20) | 8.11 (3.80) | 14.1 (2.17) | 10.1 (3.04) | 14.0 (2.18) | 7.76 (3.79) | 14.0 (2.19) | 7.39 (3.88) | 14.0 (2.19) | 7.19 (3.90) | 14.0 (2.19) | 9.06 (3.36) | 14.0 (2.19) | 6.72 (3.91) | 14.0 (2.19) | 8.04 (3.90) |
| **Age at diagnosis [mean (SD)]** | - | - | 65.0 (9.36) | - | 74.7 (5.26) | - | 67.1 (8.72) | - | 63.6 (9.50) | - | 62.5 (9.68) | - | 72.0 (6.26) | - | 63.5 (8.81) | - | 66.2 (9.55) |
| **Benzene [mean (SD)], μg/m^3^** | 0.72 (0.20) | 0.72 (0.20) | 0.73 (0.19) | 0.72 (0.20) | 0.71 (0.19) | 0.72 (0.20) | 0.73 (0.20) | 0.72 (0.20) | 0.73 (0.19) | 0.72 (0.20) | 0.73 (0.20) | 0.72 (0.20) | 0.71 (0.19) | 0.72 (0.20) | 0.74 (0.21) | 0.72 (0.20) | 0.77 (0.23) |
| Low | 72091 (25.0) | 69593 (25.2) | 2498 (20.3) | 70861 (25.0) | 1230 (25.1) | 71633 (25.0) | 458 (22.9) | 69365 (25.1) | 2726 (22.1) | 71318 (25.0) | 773 (22.9) | 71599 (25.0) | 492 (25.2) | 70788 (25.1) | 1303 (22.0) | 71967 (25.0) | 124 (22.3) |
| Median | 144071 (50.0) | 137422 (49.8) | 6649 (53.9) | 141519 (50.0) | 2552 (52.1) | 143056 (50.0) | 1015 (50.7) | 137582 (49.9) | 6489 (52.7) | 142368 (50.0) | 1703 (50.4) | 143031 (50.0) | 1040 (53.2) | 141141 (50.0) | 2930 (49.5) | 143834 (50.0) | 237 (42.6) |
| High | 72018 (25.0) | 68836 (25.0) | 3182 (25.8) | 70903 (25.0) | 1115 (22.8) | 71488 (25.0) | 530 (26.5) | 68914 (25.0) | 3104 (25.2) | 71116 (25.0) | 902 (26.7) | 71595 (25.0) | 423 (21.6) | 70333 (24.9) | 1685 (28.5) | 71823 (25.0) | 195 (35.1) |
| **Age at recruitment [mean (SD)]** | 56.5 (8.16) | 56.5 (8.15) | 56.9 (8.33) | 56.3 (8.14) | 64.6 (4.38) | 56.5 (8.16) | 59.3 (7.61) | 56.5 (8.15) | 56.2 (8.39) | 56.5 (8.16) | 55.3 (8.53) | 56.4 (8.16) | 63.0 (5.36) | 56.5 (8.17) | 56.8 (7.89) | 56.5 (8.16) | 58.2 (8.38) |
| **Sex [n (%)]** |  |  |  |  |  |  |  |  |  |  |  |  |  |  |  |  |  |
| Male | 138296 (48.0) | 133868 (48.5) | 4428 (35.9) | 135644 (47.9) | 2652 (54.2) | 137169 (47.9) | 1127 (56.3) | 133289 (48.3) | 5007 (40.6) | 137244 (48.2) | 1052 (31.1) | 137024 (47.9) | 1272 (65.1) | 134517 (47.7) | 3779 (63.9) | 138008 (48.0) | 288 (51.8) |
| Female | 149884 (52.0) | 141983 (51.5) | 7901 (64.1) | 147639 (52.1) | 2245 (45.8) | 149008 (52.1) | 876 (43.7) | 142572 (51.7) | 7312 (59.4) | 147558 (51.8) | 2326 (68.9) | 149201 (52.1) | 683 (34.9) | 147745 (52.3) | 2139 (36.1) | 149616 (52.0) | 268 (48.2) |
| **Qualifications** |  |  |  |  |  |  |  |  |  |  |  |  |  |  |  |  |  |
| Higher education | 111303 (38.6) | 107447 (39.0) | 3856 (31.3) | 109975 (38.8) | 1328 (27.1) | 110643 (38.7) | 660 (33.0) | 107549 (39.0) | 3754 (30.5) | 110036 (38.6) | 1267 (37.5) | 110639 (38.7) | 664 (34.0) | 109323 (38.7) | 1980 (33.5) | 111117 (38.6) | 186 (33.5) |
| Lower education | 176877 (61.4) | 168404 (61.0) | 8473 (68.7) | 173308 (61.2) | 3569 (72.9) | 175534 (61.3) | 1343 (67.0) | 168312 (61.0) | 8565 (69.5) | 174766 (61.4) | 2111 (62.5) | 175586 (61.3) | 1291 (66.0) | 172939 (61.3) | 3938 (66.5) | 176507 (61.4) | 370 (66.5) |
| **Body mass index [mean (SD)], Kg/m^2^** | 27.4 (4.69) | 27.3 (4.67) | 27.7 (5.15) | 27.4 (4.69) | 27.7 (4.74) | 27.4 (4.69) | 27.9 (4.81) | 27.3 (4.65) | 28.4 (5.40) | 27.4 (4.69) | 27.4 (4.96) | 27.4 (4.69) | 27.7 (4.39) | 27.3 (4.61) | 31.0 (6.46) | 27.4 (4.69) | 28.0 (4.74) |
| **CCI [mean (SD)], μg/m^3^** | 1.67 (1.30) | 1.66 (1.30) | 1.82 (1.35) | 1.65 (1.29) | 2.79 (1.33) | 1.67 (1.30) | 2.27 (1.45) | 1.66 (1.29) | 1.85 (1.44) | 1.67 (1.30) | 1.63 (1.35) | 1.67 (1.30) | 2.45 (1.22) | 1.66 (1.29) | 2.00 (1.50) | 1.67 (1.30) | 2.11 (1.54) |
| **Alcohol consumption** |  |  |  |  |  |  |  |  |  |  |  |  |  |  |  |  |  |
| Drink moderately | 96692 (33.6) | 93154 (33.8) | 3538 (28.7) | 95138 (33.6) | 1554 (31.7) | 96065 (33.6) | 627 (31.3) | 93248 (33.8) | 3444 (28.0) | 95727 (33.6) | 965 (28.6) | 95942 (33.5) | 750 (38.4) | 94770 (33.6) | 1922 (32.5) | 96540 (33.6) | 152 (27.3) |
| Inappropriate drinking | 191488 (66.4) | 182697 (66.2) | 8791 (71.3) | 188145 (66.4) | 3343 (68.3) | 190112 (66.4) | 1376 (68.7) | 182613 (66.2) | 8875 (72.0) | 189075 (66.4) | 2413 (71.4) | 190283 (66.5) | 1205 (61.6) | 187492 (66.4) | 3996 (67.5) | 191084 (66.4) | 404 (72.7) |
| **Ever smoked** |  |  |  |  |  |  |  |  |  |  |  |  |  |  |  |  |  |
| No | 117425 (40.7) | 112769 (40.9) | 4656 (37.8) | 115646 (40.8) | 1779 (36.3) | 116686 (40.8) | 739 (36.9) | 113134 (41.0) | 4291 (34.8) | 115977 (40.7) | 1448 (42.9) | 116664 (40.8) | 761 (38.9) | 115329 (40.9) | 2096 (35.4) | 117225 (40.8) | 200 (36.0) |
| Yes | 170755 (59.3) | 163082 (59.1) | 7673 (62.2) | 167637 (59.2) | 3118 (63.7) | 169491 (59.2) | 1264 (63.1) | 162727 (59.0) | 8028 (65.2) | 168825 (59.3) | 1930 (57.1) | 169561 (59.2) | 1194 (61.1) | 166933 (59.1) | 3822 (64.6) | 170399 (59.2) | 356 (64.0) |
| **Moderate sleep** |  |  |  |  |  |  |  |  |  |  |  |  |  |  |  |  |  |
| Moderate | 213254 (74.0) | 204841 (74.3) | 8413 (68.2) | 209737 (74.0) | 3517 (71.8) | 211866 (74.0) | 1388 (69.3) | 205099 (74.3) | 8155 (66.2) | 210867 (74.0) | 2387 (70.7) | 211774 (74.0) | 1480 (75.7) | 209546 (74.2) | 3708 (62.7) | 212886 (74.0) | 368 (66.2) |
| Improper | 74926 (26.0) | 71010 (25.7) | 3916 (31.8) | 73546 (26.0) | 1380 (28.2) | 74311 (26.0) | 615 (30.7) | 70762 (25.7) | 4164 (33.8) | 73935 (26.0) | 991 (29.3) | 74451 (26.0) | 475 (24.3) | 72716 (25.8) | 2210 (37.3) | 74738 (26.0) | 188 (33.8) |
| **Moderate physical activity [mean (SD)], minutes/week** | 929 (1202) | 928 (1201) | 952 (1240) | 927 (1201) | 1065 (1294) | 929 (1202) | 990 (1272) | 928 (1200) | 944 (1247) | 929 (1202) | 913 (1197) | 929 (1202) | 941 (1202) | 931 (1203) | 846 (1168) | 929 (1202) | 1055 (1332) |
| **Healthy diet score [mean (SD)]** | 3.98 (1.09) | 3.97 (1.09) | 4.01 (1.10) | 3.97 (1.09) | 4.04 (1.07) | 3.98 (1.09) | 3.94 (1.11) | 3.98 (1.09) | 3.95 (1.11) | 3.97 (1.09) | 4.09 (1.10) | 3.98 (1.09) | 3.99 (1.06) | 3.98 (1.09) | 3.82 (1.07) | 3.98 (1.09) | 3.90 (1.08) |
| **Inverse distance to the nearest major road [mean (SD)], 1/metres** | 0.01 (0.01) | 0.01 (0.01) | 0.01 (0.01) | 0.01 (0.01) | 0.01 (0.01) | 0.01 (0.01) | 0.01 (0.03) | 0.01 (0.01) | 0.01 (0.01) | 0.01 (0.01) | 0.01 (0.01) | 0.01 (0.01) | 0.01 (0.01) | 0.01 (0.01) | 0.01 (0.01) | 0.01 (0.01) | 0.01 (0.01) |
| **Noise pollution [mean (SD)], dB** | 56.0 (4.27) | 56.0 (4.27) | 56.1 (4.26) | 56.0 (4.27) | 56.0 (4.23) | 56.0 (4.27) | 56.2 (4.44) | 56.0 (4.27) | 56.1 (4.32) | 56.0 (4.27) | 56.2 (4.31) | 56.0 (4.27) | 56.0 (4.24) | 56.0 (4.27) | 56.2 (4.43) | 56.0 (4.27) | 56.3 (4.48) |

MDD, major depressive disorder; PD, Parkinson’s disease; SD, Sleep disorders; SSD, schizophrenia spectrum disorders.

# **Supplementary Table 8. Association between ambient benzene exposure and brain disorders.**

| **Outcome** | **Benzene** | **Model 1** | | **Model 2** | | **Model 3** | |
| --- | --- | --- | --- | --- | --- | --- | --- |
|  |  | **HR (95% CI)** | **P-value** | **HR (95% CI)** | **P-value** | **HR (95% CI)** | **P-value** |
| Anxiety | Per IQR | 1.10 (1.07, 1.12) | 6.33×10^-19^ | 1.11 (1.09, 1.14) | 1.02×10^-24^ | 1.10 (1.08, 1.13) | 3.09×10^-20^ |
|  | Low | 1.00 (reference) | - | 1.00 (reference) | - | 1.00 (reference) | - |
|  | Moderate | 1.35 (1.29, 1.41) | 1.57×10^-37^ | 1.34 (1.28, 1.40) | 7.04×10^-35^ | 1.33 (1.27, 1.39) | 2.02×10^-33^ |
|  | High | 1.32 (1.25, 1.39) | 3.62×10^-25^ | 1.35 (1.28, 1.42) | 1.08×10^-28^ | 1.32 (1.25, 1.39) | 1.55×10^-24^ |
| Dementia | Per IQR | 0.98 (0.95, 1.01) | 2.66×10^-01^ | 1.07 (1.03, 1.10) | 2.21×10^-04^ | 1.06 (1.02, 1.09) | 1.47×10^-03^ |
|  | Low | 1.00 (reference) | - | 1.00 (reference) | - | 1.00 (reference) | - |
|  | Moderate | 1.06 (0.99, 1.13) | 1.23×10^-01^ | 1.08 (1.01, 1.16) | 2.12×10^-02^ | 1.08 (1.01, 1.15) | 3.11×10^-02^ |
|  | High | 0.96 (0.89, 1.04) | 3.61×10^-01^ | 1.16 (1.07, 1.26) | 4.26×10^-04^ | 1.14 (1.05, 1.23) | 2.13×10^-03^ |
| Epilepsy | Per IQR | 1.06 (1.00, 1.11) | 3.70×10^-02^ | 1.09 (1.03, 1.14) | 1.56×10^-03^ | 1.08 (1.02, 1.13) | 6.72×10^-03^ |
|  | Low | 1.00 (reference) | - | 1.00 (reference) | - | 1.00 (reference) | - |
|  | Moderate | 1.12 (1.00, 1.25) | 5.15×10^-02^ | 1.12 (1.00, 1.25) | 4.66×10^-02^ | 1.11 (1.00, 1.24) | 6.00×10^-02^ |
|  | High | 1.19 (1.05, 1.35) | 7.10×10^-03^ | 1.26 (1.11, 1.43) | 2.70×10^-04^ | 1.23 (1.09, 1.40) | 1.08×10^-03^ |
| MDD | Per IQR | 1.05 (1.03, 1.07) | 3.39×10^-06^ | 1.05 (1.03, 1.08) | 1.36×10^-06^ | 1.04 (1.01, 1.06) | 1.22×10^-03^ |
|  | Low | 1.00 (reference) | - | 1.00 (reference) | - | 1.00 (reference) | - |
|  | Moderate | 1.20 (1.15, 1.26) | 6.55×10^-16^ | 1.17 (1.12, 1.23) | 2.16×10^-12^ | 1.16 (1.11, 1.22) | 4.54×10^-11^ |
|  | High | 1.17 (1.11, 1.23) | 3.55×10^-09^ | 1.16 (1.10, 1.22) | 3.15×10^-08^ | 1.12 (1.06, 1.18) | 2.64×10^-05^ |
| Migraine | Per IQR | 1.08 (1.03, 1.12) | 2.72×10^-04^ | 1.06 (1.02, 1.10) | 2.86×10^-03^ | 1.05 (1.01, 1.09) | 1.44×10^-02^ |
|  | Low | 1.00 (reference) | - | 1.00 (reference) | - | 1.00 (reference) | - |
|  | Moderate | 1.11 (1.02, 1.21) | 1.59×10^-02^ | 1.10 (1.01, 1.20) | 2.90×10^-02^ | 1.09 (1.00, 1.19) | 4.02×10^-02^ |
|  | High | 1.19 (1.08, 1.31) | 3.33×10^-04^ | 1.16 (1.05, 1.27) | 3.10×10^-03^ | 1.13 (1.03, 1.25) | 1.27×10^-02^ |
| PD | Per IQR | 0.96 (0.91, 1.01) | 1.41×10^-01^ | 1.02 (0.97, 1.08) | 3.74×10^-01^ | 1.03 (0.97, 1.09) | 3.34×10^-01^ |
|  | Low | 1.00 (reference) | - | 1.00 (reference) | - | 1.00 (reference) | - |
|  | Moderate | 1.07 (0.96, 1.19) | 2.38×10^-01^ | 1.10 (0.99, 1.22) | 8.66×10^-02^ | 1.10 (0.99, 1.23) | 7.95×10^-02^ |
|  | High | 0.89 (0.78, 1.01) | 8.14×10^-02^ | 1.03 (0.91, 1.17) | 6.44×10^-01^ | 1.04 (0.91, 1.18) | 6.04×10^-01^ |
| SD | Per IQR | 1.14 (1.11, 1.17) | 6.80×10^-19^ | 1.14 (1.11, 1.18) | 1.57×10^-18^ | 1.13 (1.10, 1.16) | 2.55×10^-15^ |
|  | Low | 1.00 (reference) | - | 1.00 (reference) | - | 1.00 (reference) | - |
|  | Moderate | 1.13 (1.06, 1.21) | 2.19×10^-04^ | 1.10 (1.03, 1.17) | 4.14×10^-03^ | 1.09 (1.02, 1.16) | 9.14×10^-03^ |
|  | High | 1.32 (1.23, 1.42) | 4.98×10^-14^ | 1.29 (1.20, 1.38) | 9.19×10^-12^ | 1.25 (1.17, 1.35) | 1.21×10^-09^ |
| SSD | Per IQR | 1.32 (1.21, 1.44) | 7.49×10^-10^ | 1.35 (1.24, 1.48) | 3.56×10^-11^ | 1.33 (1.22, 1.46) | 7.69×10^-10^ |
|  | Low | 1.00 (reference) | - | 1.00 (reference) | - | 1.00 (reference) | - |
|  | Moderate | 0.97 (0.78, 1.20) | 7.49×10^-01^ | 0.96 (0.77, 1.20) | 7.30×10^-01^ | 0.95 (0.77, 1.18) | 6.57×10^-01^ |
|  | High | 1.63 (1.30, 2.05) | 1.97×10^-05^ | 1.71 (1.36, 2.14) | 3.67×10^-06^ | 1.64 (1.31, 2.06) | 2.03×10^-05^ |

Benzene exposure was categorised into three monotonically increasing exposure levels based on quartiles: low (≤0.58 μg/m^3^), moderate (0.58-0.82 μg/m^3^), and high (>0.82 μg/m^3^). Hazard ratios were estimated using Cox proportional hazards regression. Two-sided Z-tests were used to assess statistical significance. Model 1: Unadjusted model. Model 2: Adjusted for age, sex, qualifications, BMI, and Charlson Comorbidity Index (CCI). Model 3: Additionally adjusted for occupational benzene exposure, alcohol consumption, smoking history, moderate sleep, moderate physical activity, healthy diet score, proximity to major roads, and noise pollution. MDD, major depressive disorder; PD, Parkinson’s disease; SD, Sleep disorders; SSD, schizophrenia spectrum disorders.

# **Supplementary Table 9. Sensitivity analysis of the associations between ambient benzene exposure and brain disorders using time-dependent Cox regression models.**

| **Outcome** | **Lag 0** | | **Lag 1** | | **Lag 2** | | **Lag 3** | |
| --- | --- | --- | --- | --- | --- | --- | --- | --- |
|  | **HR (95% CI)** | **P-value** | **HR (95% CI)** | **P-value** | **HR (95% CI)** | **P-value** | **HR (95% CI)** | **P-value** |
| Anxiety | 1.14 (1.11, 1.17) | 1.69×10^-25^ | 1.11 (1.08, 1.14) | 7.45×10^-17^ | 1.12 (1.10, 1.15) | 1.03×10^-20^ | 1.14 (1.11, 1.17) | 1.48×10^-25^ |
| Dementia | 0.98 (0.94, 1.02) | 4.28×10^-01^ | 0.97 (0.93, 1.01) | 1.54×10^-01^ | 1.03 (0.99, 1.07) | 1.45×10^-01^ | 1.10 (1.05, 1.15) | 1.09×10^-05^ |
| Epilepsy | 1.07 (1.01, 1.14) | 3.20×10^-02^ | 1.05 (0.99, 1.12) | 1.06×10^-01^ | 1.08 (1.01, 1.14) | 1.86×10^-02^ | 1.12 (1.05, 1.19) | 4.03×10^-04^ |
| MDD | 1.04 (1.01, 1.07) | 2.02×10^-03^ | 1.02 (0.99, 1.04) | 2.31×10^-01^ | 1.03 (1.00, 1.05) | 2.66×10^-02^ | 1.05 (1.03, 1.08) | 5.40×10^-05^ |
| Migraine | 1.08 (1.03, 1.13) | 1.17×10^-03^ | 1.07 (1.02, 1.12) | 4.40×10^-03^ | 1.06 (1.02, 1.11) | 8.95×10^-03^ | 1.06 (1.01, 1.11) | 1.19×10^-02^ |
| PD | 1.02 (0.96, 1.09) | 5.22×10^-01^ | 0.99 (0.93, 1.05) | 7.42×10^-01^ | 1.00 (0.94, 1.07) | 9.40×10^-01^ | 1.02 (0.96, 1.09) | 4.77×10^-01^ |
| SD | 1.13 (1.09, 1.17) | 1.29×10^-12^ | 1.10 (1.06, 1.14) | 1.21×10^-07^ | 1.11 (1.07, 1.15) | 5.63×10^-09^ | 1.12 (1.09, 1.16) | 1.31×10^-11^ |
| SSD | 1.22 (1.09, 1.36) | 5.61×10^-04^ | 1.18 (1.06, 1.32) | 3.47×10^-03^ | 1.21 (1.08, 1.36) | 7.34×10^-04^ | 1.26 (1.13, 1.41) | 5.53×10^-05^ |

Hazard ratios were estimated per interquartile-range (IQR) increase in benzene exposure using time-dependent Cox proportional hazards models. Lag 0, lag 1, lag 2, and lag 3 represent benzene exposure in the same year, and 1, 2, and 3 years before, respectively. The corresponding IQRs were 0.1884766 μg/m³ for lag 0, 0.1887647 μg/m³ for lag 1, 0.19493 μg/m³ for lag 2, and 0.2055648 μg/m³ for lag 3. All models were fully adjusted for age, sex, qualifications, BMI, Charlson Comorbidity Index (CCI), occupational benzene exposure, alcohol consumption, smoking history, moderate sleep, moderate physical activity, healthy diet score, proximity to major roads, and noise pollution. Two-sided Z-tests were used to assess statistical significance. MDD, major depressive disorder; PD, Parkinson’s disease; SD, sleep disorders; SSD, schizophrenia spectrum disorders.

# **Supplementary Table 10. Risk of incident brain disorders according to categories of benzene exposure within each genetic risk stratum.**

| **Outcome** | **Benzene** | **Genetic risk** | **n / N** | **HR (95% CI)** | **P-value** |
| --- | --- | --- | --- | --- | --- |
| Anxiety | Low | Lower | 603 / 18251 | 1.00 (reference) | - |
|  | Low | Intermediate | 1244 / 36371 | 1.03 (0.94, 1.14) | 5.07×10^-01^ |
|  | Low | Higher | 651 / 17469 | 1.13 (1.01, 1.26) | 3.18×10^-02^ |
|  | Moderate | Lower | 1609 / 36495 | 1.33 (1.21, 1.46) | 3.19×10^-09^ |
|  | Moderate | Intermediate | 3298 / 72294 | 1.37 (1.26, 1.50) | 7.70×10^-13^ |
|  | Moderate | Higher | 1742 / 35282 | 1.49 (1.36, 1.63) | 3.58×10^-17^ |
|  | High | Lower | 758 / 17296 | 1.38 (1.24, 1.53) | 5.49×10^-09^ |
|  | High | Intermediate | 1561 / 35424 | 1.38 (1.26, 1.52) | 2.21×10^-11^ |
|  | High | Higher | 863 / 19298 | 1.39 (1.25, 1.54) | 7.61×10^-10^ |
| Dementia | Low | Lower | 210 / 18542 | 1.00 (reference) | - |
|  | Low | Intermediate | 586 / 36021 | 1.45 (1.24, 1.69) | 4.37×10^-06^ |
|  | Low | Higher | 434 / 17528 | 2.28 (1.93, 2.69) | 1.24×10^-22^ |
|  | Moderate | Lower | 458 / 36823 | 1.13 (0.96, 1.33) | 1.51×10^-01^ |
|  | Moderate | Intermediate | 1181 / 71899 | 1.52 (1.32, 1.77) | 1.79×10^-08^ |
|  | Moderate | Higher | 913 / 35349 | 2.45 (2.11, 2.85) | 1.08×10^-31^ |
|  | High | Lower | 190 / 16679 | 1.20 (0.98, 1.46) | 7.07×10^-02^ |
|  | High | Intermediate | 526 / 36168 | 1.65 (1.40, 1.93) | 1.16×10^-09^ |
|  | High | Higher | 399 / 19171 | 2.43 (2.05, 2.87) | 3.02×10^-25^ |
| Epilepsy | Low | Lower | 108 / 18012 | 1.00 (reference) | - |
|  | Low | Intermediate | 236 / 35508 | 1.10 (0.88, 1.38) | 4.16×10^-01^ |
|  | Low | Higher | 114 / 18571 | 1.01 (0.78, 1.32) | 9.26×10^-01^ |
|  | Moderate | Lower | 227 / 35991 | 1.05 (0.84, 1.32) | 6.71×10^-01^ |
|  | Moderate | Intermediate | 504 / 71731 | 1.17 (0.95, 1.44) | 1.45×10^-01^ |
|  | Moderate | Higher | 284 / 36349 | 1.30 (1.04, 1.62) | 2.16×10^-02^ |
|  | High | Lower | 137 / 18036 | 1.34 (1.04, 1.72) | 2.38×10^-02^ |
|  | High | Intermediate | 255 / 36869 | 1.23 (0.98, 1.54) | 7.66×10^-02^ |
|  | High | Higher | 138 / 17113 | 1.42 (1.10, 1.82) | 6.95×10^-03^ |
| MDD | Low | Lower | 518 / 19527 | 1.00 (reference) | - |
|  | Low | Intermediate | 1405 / 36882 | 1.39 (1.26, 1.54) | 1.15×10^-10^ |
|  | Low | Higher | 803 / 15682 | 1.79 (1.61, 2.00) | 3.35×10^-25^ |
|  | Moderate | Lower | 1257 / 36547 | 1.26 (1.14, 1.39) | 1.07×10^-05^ |
|  | Moderate | Intermediate | 3180 / 73285 | 1.55 (1.41, 1.70) | 3.30×10^-20^ |
|  | Moderate | Higher | 2052 / 34239 | 2.08 (1.89, 2.29) | 4.34×10^-50^ |
|  | High | Lower | 511 / 15972 | 1.20 (1.06, 1.35) | 4.05×10^-03^ |
|  | High | Intermediate | 1471 / 33930 | 1.58 (1.43, 1.75) | 3.75×10^-19^ |
|  | High | Higher | 1122 / 22116 | 1.73 (1.56, 1.92) | 1.18×10^-24^ |
| Migraine | Low | Lower | 179 / 19099 | 1.00 (reference) | - |
|  | Low | Intermediate | 407 / 37086 | 1.17 (0.98, 1.40) | 7.82×10^-02^ |
|  | Low | Higher | 187 / 15906 | 1.23 (1.00, 1.51) | 4.64×10^-02^ |
|  | Moderate | Lower | 375 / 36965 | 1.08 (0.90, 1.29) | 4.24×10^-01^ |
|  | Moderate | Intermediate | 855 / 73465 | 1.23 (1.05, 1.45) | 1.18×10^-02^ |
|  | Moderate | Higher | 473 / 33641 | 1.46 (1.23, 1.74) | 1.61×10^-05^ |
|  | High | Lower | 151 / 15985 | 0.99 (0.80, 1.23) | 9.48×10^-01^ |
|  | High | Intermediate | 412 / 33527 | 1.28 (1.08, 1.53) | 5.47×10^-03^ |
|  | High | Higher | 339 / 22506 | 1.50 (1.25, 1.80) | 1.21×10^-05^ |
| PD | Low | Lower | 80 / 18375 | 1.00 (reference) | - |
|  | Low | Intermediate | 215 / 36249 | 1.37 (1.06, 1.77) | 1.67×10^-02^ |
|  | Low | Higher | 197 / 17467 | 2.64 (2.04, 3.43) | 2.26×10^-13^ |
|  | Moderate | Lower | 166 / 36392 | 1.09 (0.84, 1.42) | 5.24×10^-01^ |
|  | Moderate | Intermediate | 498 / 72136 | 1.66 (1.31, 2.11) | 2.37×10^-05^ |
|  | Moderate | Higher | 376 / 35543 | 2.56 (2.01, 3.26) | 2.11×10^-14^ |
|  | High | Lower | 77 / 17278 | 1.22 (0.89, 1.67) | 2.06×10^-01^ |
|  | High | Intermediate | 197 / 35705 | 1.53 (1.18, 1.98) | 1.48×10^-03^ |
|  | High | Higher | 149 / 19035 | 2.25 (1.71, 2.95) | 5.96×10^-09^ |
| SD | Low | Lower | 252 / 18336 | 1.00 (reference) | - |
|  | Low | Intermediate | 654 / 35770 | 1.24 (1.07, 1.43) | 3.67×10^-03^ |
|  | Low | Higher | 397 / 17985 | 1.38 (1.17, 1.61) | 7.60×10^-05^ |
|  | Moderate | Lower | 601 / 36420 | 1.15 (0.99, 1.33) | 6.44×10^-02^ |
|  | Moderate | Intermediate | 1411 / 71623 | 1.29 (1.13, 1.47) | 2.10×10^-04^ |
|  | Moderate | Higher | 918 / 36028 | 1.58 (1.38, 1.82) | 1.25×10^-10^ |
|  | High | Lower | 310 / 17287 | 1.28 (1.08, 1.51) | 3.67×10^-03^ |
|  | High | Intermediate | 858 / 36707 | 1.52 (1.32, 1.75) | 4.87×10^-09^ |
|  | High | Higher | 517 / 18024 | 1.76 (1.52, 2.05) | 2.03×10^-13^ |
| SSD | Low | Lower | 32 / 18801 | 1.00 (reference) | - |
|  | Low | Intermediate | 62 / 36272 | 1.00 (0.65, 1.53) | 9.99×10^-01^ |
|  | Low | Higher | 30 / 17018 | 1.03 (0.62, 1.69) | 9.14×10^-01^ |
|  | Moderate | Lower | 48 / 36998 | 0.76 (0.48, 1.19) | 2.26×10^-01^ |
|  | Moderate | Intermediate | 111 / 72320 | 0.89 (0.60, 1.32) | 5.64×10^-01^ |
|  | Moderate | Higher | 78 / 34753 | 1.32 (0.87, 1.99) | 1.89×10^-01^ |
|  | High | Lower | 26 / 16241 | 0.97 (0.58, 1.63) | 9.08×10^-01^ |
|  | High | Intermediate | 104 / 35495 | 1.80 (1.21, 2.67) | 3.93×10^-03^ |
|  | High | Higher | 65 / 20282 | 1.97 (1.29, 3.01) | 1.82×10^-03^ |

Benzene exposure was categorised into three monotonically increasing exposure levels based on quartiles: low (≤0.58 μg/m^3^), moderate (0.58-0.82 μg/m^3^), and high (>0.82 μg/m^3^). Genetic risk was classified into lower, intermediate, and higher groups according to the distribution of the polygenic risk score (PRS): lower (≤Q1), intermediate (Q1-Q3), and higher (>Q3). Hazard ratios (HRs) were estimated using Cox proportional hazards models with the low benzene-low genetic risk group as the reference, adjusted for age, sex, qualifications, BMI, Charlson Comorbidity Index (CCI), occupational benzene exposure, alcohol consumption, smoking history, moderate sleep, moderate physical activity, healthy diet score, proximity to major roads, and noise pollution. Two-sided Z-tests were used to assess statistical significance. N, number of individuals at risk; n, number of cases. MDD, major depressive disorder; PD, Parkinson’s disease; SD, Sleep disorders; SSD, schizophrenia spectrum disorders.

# **Supplementary Table 11. Additive interaction between benzene exposure and genetic risk on brain disorders.**

| **Outcome** | **Measures** | **Estimates** | **CI.ll** | **CI.ul** | **P-value** |
| --- | --- | --- | --- | --- | --- |
| Anxiety disorders | Multiplicative scale | 0.89345 | 0.77081 | 1.03561 | 1.35×10^-01^ |
|  | RERI | -0.11701 | -0.30047 | 0.06645 | 8.94×10^-01^ |
|  | AP | -0.08431 | -0.21637 | 0.04775 | 1.05×10^-01^ |
|  | SI | 0.76823 | 0.53090 | 1.11163 | 9.19×10^-01^ |
| Dementia | Multiplicative scale | 0.88991 | 0.70090 | 1.12988 | 3.38×10^-01^ |
|  | RERI | -0.04666 | -0.43668 | 0.34335 | 5.93×10^-01^ |
|  | AP | -0.01921 | -0.17999 | 0.14158 | 4.07×10^-01^ |
|  | SI | 0.96839 | 0.74297 | 1.26220 | 5.94×10^-01^ |
| Epilepsy | Multiplicative scale | 1.04453 | 0.73329 | 1.48788 | 8.09×10^-01^ |
|  | RERI | 0.06462 | -0.34984 | 0.47909 | 3.80×10^-01^ |
|  | AP | 0.04564 | -0.24660 | 0.33788 | 3.80×10^-01^ |
|  | SI | 1.18390 | 0.36353 | 3.85560 | 3.90×10^-01^ |
| MDD | Multiplicative scale | 0.80530 | 0.69157 | 0.93772 | 5.31×10^-03^ |
|  | RERI | -0.26187 | -0.48175 | -0.04199 | 9.90×10^-01^ |
|  | AP | -0.15139 | -0.27720 | -0.02559 | 9.17×10^-03^ |
|  | SI | 0.73591 | 0.58944 | 0.91879 | 9.97×10^-01^ |
| Migraine | Multiplicative scale | 1.22875 | 0.92781 | 1.62729 | 1.51×10^-01^ |
|  | RERI | 0.27804 | -0.02758 | 0.58367 | 3.73×10^-02^ |
|  | AP | 0.18506 | -0.01999 | 0.39010 | 3.85×10^-02^ |
|  | SI | 2.23889 | 0.50002 | 10.02485 | 1.46×10^-01^ |
| PD | Multiplicative scale | 0.69374 | 0.47517 | 1.01286 | 5.83×10^-02^ |
|  | RERI | -0.62271 | -1.30689 | 0.06147 | 9.63×10^-01^ |
|  | AP | -0.27735 | -0.58354 | 0.02883 | 3.79×10^-02^ |
|  | SI | 0.66663 | 0.45349 | 0.97994 | 9.80×10^-01^ |
| SD | Multiplicative scale | 1.00102 | 0.81016 | 1.23683 | 9.93×10^-01^ |
|  | RERI | 0.10704 | -0.16483 | 0.37891 | 2.20×10^-01^ |
|  | AP | 0.06072 | -0.09387 | 0.21530 | 2.21×10^-01^ |
|  | SI | 1.16320 | 0.76504 | 1.76858 | 2.40×10^-01^ |
| SSD | Multiplicative scale | 1.97421 | 1.00544 | 3.87641 | 4.82×10^-02^ |
|  | RERI | 0.97018 | 0.25881 | 1.68154 | 3.76×10^-03^ |
|  | AP | 0.49304 | 0.15084 | 0.83524 | 2.37×10^-03^ |
|  | SI | - | - | - | - |

Benzene exposure was categorised into three monotonically increasing exposure levels based on quartiles: low (≤0.58 μg/m^3^), moderate (0.58-0.82 μg/m^3^), and high (>0.82 μg/m^3^). Genetic risk was classified into lower, intermediate, and higher groups according to the distribution of the polygenic risk score (PRS): lower (≤Q1), intermediate (Q1-Q3), and higher (>Q3). Estimates and 95% confidence intervals were calculated using the *interactionR* package, adjusted for age, sex, qualifications, BMI, Charlson Comorbidity Index (CCI), occupational benzene exposure, alcohol consumption, smoking history, moderate sleep, moderate physical activity, healthy diet score, proximity to major roads, and noise pollution. Two-sided Z-tests were used to assess statistical significance. N, number of individuals at risk; n, number of cases. RERI, relative excess risk due to interaction; AP, attributable proportion due to interaction; SI, synergy index. MDD, major depressive disorder; PD, Parkinson’s disease; SD, Sleep disorders; SSD, schizophrenia spectrum disorders.

# **Supplementary Table 12. Mediation analysis results for proteins in the association between benzene exposure and brain disorders.**

| **Proteins** | **Total effect** | | **Natural direct effect** | | **Natural indirect effect** | | **Proportion of mediation** | |
| --- | --- | --- | --- | --- | --- | --- | --- | --- |
|  | **Estimate (95% CI)** | **P-value** | **Estimate (95% CI)** | **P-value** | **Estimate (95% CI)** | **P-value** | **Estimate (%, 95% CI)** | **P-value** |
| GDF15 | 128.7 (112.5, 145.8) | 0.000 | 125.4 (109.5, 142.2) | 0.000 | 102.6 (101.8, 103.6) | 0.000 | 11.4 (6.9, 24.7) | 0.000 |
| DTNB | 129.5 (113.6, 149.4) | 0.000 | 126.6 (111.2, 146.3) | 0.002 | 102.3 (101.4, 103.3) | 0.000 | 10.1 (5.4, 20.7) | 0.000 |
| ANGPT2 | 129.4 (112.8, 148.2) | 0.000 | 126.9 (110.6, 145.2) | 0.000 | 102.0 (101.3, 102.9) | 0.000 | 8.6 (4.9, 18.4) | 0.000 |
| MMP12 | 129.6 (111.4, 149.2) | 0.000 | 127.3 (109.3, 146.8) | 0.000 | 101.9 (101.2, 102.6) | 0.000 | 8.1 (4.5, 18.9) | 0.000 |
| IL15 | 129.2 (112.5, 147.9) | 0.000 | 126.8 (110.3, 145.5) | 0.000 | 101.8 (101.0, 102.7) | 0.002 | 8.0 (3.8, 19.2) | 0.002 |
| CCL15 | 129.3 (113.7, 149.4) | 0.000 | 127.0 (111.9, 146.5) | 0.000 | 101.8 (101.0, 102.6) | 0.000 | 7.8 (4.0, 15.5) | 0.000 |
| IFI30 | 129.7 (113.3, 149.5) | 0.000 | 127.4 (111.3, 146.9) | 0.000 | 101.8 (101.1, 102.6) | 0.000 | 7.7 (4.3, 17.0) | 0.000 |
| CDCP1 | 129.6 (113.6, 148.8) | 0.000 | 127.3 (111.8, 146.6) | 0.000 | 101.8 (101.0, 102.6) | 0.000 | 7.7 (4.2, 16.6) | 0.000 |
| OMG | 129.4 (113.5, 148.1) | 0.004 | 127.1 (111.3, 145.7) | 0.006 | 101.8 (101.1, 102.6) | 0.000 | 7.7 (4.0, 16.7) | 0.004 |
| OGN | 129.3 (113.0, 148.0) | 0.000 | 127.1 (111.4, 145.5) | 0.000 | 101.7 (100.9, 102.5) | 0.000 | 7.3 (3.8, 15.1) | 0.000 |
| TNFRSF10A | 129.6 (113.9, 150.9) | 0.000 | 127.5 (111.9, 148.4) | 0.000 | 101.7 (101.0, 102.4) | 0.000 | 7.3 (3.8, 15.6) | 0.000 |
| FOLR2 | 129.5 (113.0, 147.5) | 0.002 | 127.3 (110.9, 145.0) | 0.002 | 101.7 (101.0, 102.5) | 0.000 | 7.3 (3.9, 15.7) | 0.002 |
| BNIP3L | 129.3 (112.7, 148.7) | 0.000 | 127.2 (111.3, 146.2) | 0.002 | 101.7 (100.7, 102.7) | 0.002 | 7.3 (2.7, 17.6) | 0.002 |
| SERPINH1 | 129.4 (112.8, 146.1) | 0.000 | 127.4 (111.0, 143.9) | 0.000 | 101.5 (100.6, 102.5) | 0.002 | 6.7 (2.4, 15.4) | 0.002 |
| PLIN3 | 129.4 (111.9, 148.9) | 0.000 | 127.5 (110.2, 146.8) | 0.000 | 101.5 (100.7, 102.5) | 0.000 | 6.6 (2.7, 15.8) | 0.000 |
| APOL1 | 129.4 (111.5, 148.3) | 0.000 | 131.7 (112.9, 151.1) | 0.000 | 98.3 (97.4, 99.2) | 0.000 | -7.6 (-17.7, -3.5) | 0.000 |
| CPM | 129.5 (113.1, 148.4) | 0.000 | 132.0 (115.8, 151.4) | 0.000 | 98.1 (97.1, 99.0) | 0.000 | -8.4 (-18.4, -3.8) | 0.000 |
| DNER | 129.4 (112.9, 148.7) | 0.000 | 132.1 (115.2, 151.9) | 0.000 | 97.9 (97.0, 98.9) | 0.000 | -9.3 (-21.3, -4.3) | 0.000 |
| ITGAM | 129.1 (111.9, 150.2) | 0.000 | 131.9 (114.4, 152.9) | 0.000 | 97.8 (97.0, 98.6) | 0.000 | -9.8 (-21.1, -5.3) | 0.000 |
| CLEC4D | 129.4 (113.6, 149.1) | 0.000 | 132.5 (116.2, 152.6) | 0.000 | 97.7 (96.8, 98.5) | 0.000 | -10.4 (-22.6, -5.6) | 0.000 |

This table presents the mediation analysis results for the top 15 proteins with the highest positive mediation proportions and the top 5 proteins with competitive (adverse) mediation effects. Estimates include the total effect, natural direct effect (NDE), natural indirect effect (NIE), and the proportion mediated, each reported with 95% confidence intervals and corresponding P-values. All analyses were adjusted for age, sex, qualifications, BMI, Charlson Comorbidity Index (CCI), occupational benzene exposure, alcohol consumption, smoking history, moderate sleep, moderate physical activity, healthy diet score, proximity to major roads, and noise pollution. Statistical significance was assessed using two-sided Z-tests.

# **Supplementary Table 13. The results of CellMarker and GTEx.**

| **Term** | **Overlap** | **P-value** | **Adjusted P-value** | **Source** | **Mdoel** |
| --- | --- | --- | --- | --- | --- |
| M2 Macrophage Lung Human | 7/33 | 8.04E-08 | 5.32E-05 | CellMarker | Model 1 |
| Microglial Cell Brain Human | 17/360 | 1.00E-06 | 3.32E-04 | CellMarker | Model 1 |
| Macrophage Lung Mouse | 5/23 | 5.47E-06 | 1.18E-03 | CellMarker | Model 1 |
| Macrophage Liver Human | 5/25 | 8.48E-06 | 1.18E-03 | CellMarker | Model 1 |
| Myeloid Cell Bone Marrow Human | 9/120 | 1.04E-05 | 1.18E-03 | CellMarker | Model 1 |
| Schwalie Et al.Nature.G2 Adipose Tissue Mouse | 11/189 | 1.25E-05 | 1.18E-03 | CellMarker | Model 1 |
| Macrophage Brain Human | 8/94 | 1.29E-05 | 1.18E-03 | CellMarker | Model 1 |
| Plasma Cell Nasopharynx Human | 7/69 | 1.43E-05 | 1.18E-03 | CellMarker | Model 1 |
| Macrophage Skin Mouse | 7/71 | 1.73E-05 | 1.27E-03 | CellMarker | Model 1 |
| Macrophage Blood Human | 5/30 | 2.17E-05 | 1.44E-03 | CellMarker | Model 1 |
| Microglial Cell Embryonic Prefrontal Cortex Human | 20/476 | 1.90E-09 | 1.44E-06 | CellMarker | Model 2 |
| Myeloid Cell Bone Marrow Human | 10/120 | 4.97E-08 | 1.89E-05 | CellMarker | Model 2 |
| Activated T Cell Undefined Human | 5/15 | 9.22E-08 | 2.28E-05 | CellMarker | Model 2 |
| Macrophage Lung Human | 8/72 | 1.20E-07 | 2.28E-05 | CellMarker | Model 2 |
| CD4+ T Cell Undefined Human | 5/18 | 2.58E-07 | 3.91E-05 | CellMarker | Model 2 |
| Natural Killer Cell Blood Human | 7/56 | 3.34E-07 | 4.22E-05 | CellMarker | Model 2 |
| Mesenchymal Stem Cell Undefined Human | 7/60 | 5.41E-07 | 5.87E-05 | CellMarker | Model 2 |
| Endothelial Cell Blood Vessel Human | 7/66 | 1.05E-06 | 9.94E-05 | CellMarker | Model 2 |
| Monocyte Fetal Kidney Human | 21/796 | 1.94E-06 | 1.35E-04 | CellMarker | Model 2 |
| Effector T Cell Undefined Human | 4/12 | 1.95E-06 | 1.35E-04 | CellMarker | Model 2 |
| Adipose - Visceral (Omentum) Female 50-59 Up | 7/100 | 1.57E-04 | 1.26E-02 | GTEx | Model 1 |
| Spleen Female 30-39 Up | 7/100 | 1.57E-04 | 1.26E-02 | GTEx | Model 1 |
| Liver Male 20-29 Up | 7/100 | 1.57E-04 | 1.26E-02 | GTEx | Model 1 |
| Adipose - Visceral (Omentum) Male 70-79 Up | 7/100 | 1.57E-04 | 1.26E-02 | GTEx | Model 1 |
| Liver Female 20-29 Up | 7/100 | 1.57E-04 | 1.26E-02 | GTEx | Model 1 |
| Stomach Male 20-29 Up | 6/86 | 4.73E-04 | 3.16E-02 | GTEx | Model 1 |
| Spleen Male 60-69 Up | 6/100 | 1.05E-03 | 3.83E-02 | GTEx | Model 1 |
| Adipose - Subcutaneous Male 40-49 Up | 6/100 | 1.05E-03 | 3.83E-02 | GTEx | Model 1 |
| Adipose - Visceral (Omentum) Male 40-49 Up | 6/100 | 1.05E-03 | 3.83E-02 | GTEx | Model 1 |
| Spleen Female 20-29 Up | 6/100 | 1.05E-03 | 3.83E-02 | GTEx | Model 1 |
| Whole Blood Female 60-69 Up | 7/100 | 1.71E-05 | 6.33E-03 | GTEx | Model 2 |
| Whole Blood Male 50-59 Up | 6/100 | 1.64E-04 | 1.21E-02 | GTEx | Model 2 |
| Lung Male 20-29 Up | 6/100 | 1.64E-04 | 1.21E-02 | GTEx | Model 2 |
| Whole Blood Male 20-29 Up | 6/100 | 1.64E-04 | 1.21E-02 | GTEx | Model 2 |
| Lung Female 20-29 Up | 6/100 | 1.64E-04 | 1.21E-02 | GTEx | Model 2 |
| Artery - Coronary Male 20-29 Up | 5/93 | 9.60E-04 | 2.14E-02 | GTEx | Model 2 |
| Artery - Tibial Male 60-69 Up | 5/100 | 1.33E-03 | 2.14E-02 | GTEx | Model 2 |
| Lung Female 60-69 Up | 5/100 | 1.33E-03 | 2.14E-02 | GTEx | Model 2 |
| Whole Blood Male 40-49 Up | 5/100 | 1.33E-03 | 2.14E-02 | GTEx | Model 2 |
| Adipose - Subcutaneous Male 40-49 Up | 5/100 | 1.33E-03 | 2.14E-02 | GTEx | Model 2 |

Model 1 presents the enrichment results for all positively mediating proteins, whereas Model 2 presents the enrichment results for all candidate proteins.

# **Supplementary Table 14. Pathway-level mediation analysis of KEGG pathways based on positively mediating proteins.**

| **KEGG Pathways** | **N** | **Total effect** | | **Natural direct effect** | | **Natural indirect effect** | | **Proportion of mediation** | |
| --- | --- | --- | --- | --- | --- | --- | --- | --- | --- |
|  |  | **Estimate (95% CI)** | **P-value** | **Estimate (95% CI)** | **P-value** | **Estimate (95% CI)** | **P-value** | **Estimate (%, 95% CI)** | **P-value** |
| PI3K-Akt signaling pathway | 14 | 129.4 (111.1, 149.1) | 0.002 | 120.7 (105.0, 139.2) | 0.012 | 107.1 (105.2, 109.2) | 0.000 | 29.4 (18.3, 58.8) | 0.002 |
| Pathways in cancer | 13 | 129.1 (111.7, 147.5) | 0.000 | 122.4 (105.7, 139.8) | 0.008 | 105.5 (103.3, 107.6) | 0.000 | 22.9 (12.9, 52.3) | 0.000 |
| MAPK signaling pathway | 7 | 129.5 (113.5, 148.1) | 0.002 | 123.2 (108.0, 141.1) | 0.002 | 105.1 (103.7, 106.6) | 0.000 | 21.3 (13.5, 42.4) | 0.002 |
| Cytokine-cytokine receptor interaction | 27 | 128.7 (112.6, 148.3) | 0.000 | 122.6 (107.6, 141.5) | 0.006 | 105.0 (102.9, 107.1) | 0.000 | 21.2 (11.2, 44.7) | 0.000 |
| Apoptosis | 6 | 129.4 (112.3, 149.1) | 0.000 | 124.2 (108.0, 142.9) | 0.000 | 104.2 (102.7, 105.7) | 0.000 | 17.7 (10.4, 39.2) | 0.000 |
| Proteoglycans in cancer | 7 | 129.1 (112.6, 148.2) | 0.000 | 124.0 (107.8, 142.6) | 0.002 | 104.1 (102.7, 105.6) | 0.000 | 17.5 (10.4, 38.9) | 0.000 |
| TNF signaling pathway | 4 | 129.3 (112.7, 148.2) | 0.000 | 124.4 (108.5, 142.8) | 0.000 | 103.9 (102.5, 105.4) | 0.000 | 16.6 (9.5, 34.4) | 0.000 |
| Rap1 signaling pathway | 5 | 129.4 (111.6, 148.7) | 0.000 | 124.5 (107.3, 143.1) | 0.000 | 103.9 (102.8, 105.1) | 0.000 | 16.4 (10.3, 36.6) | 0.000 |
| Human T-cell leukemia virus 1 infection | 6 | 129.2 (112.1, 149.2) | 0.000 | 124.5 (108.6, 143.7) | 0.000 | 103.7 (102.4, 105.0) | 0.000 | 16.0 (9.6, 32.0) | 0.000 |
| JAK-STAT signaling pathway | 7 | 129.2 (112.9, 148.5) | 0.000 | 124.6 (108.6, 143.3) | 0.002 | 103.7 (102.5, 105.0) | 0.000 | 15.9 (9.2, 33.9) | 0.000 |
| Transcriptional misregulation in cancer | 8 | 129.5 (112.1, 149.3) | 0.000 | 124.8 (108.0, 143.9) | 0.000 | 103.7 (102.6, 104.9) | 0.000 | 15.8 (9.2, 34.1) | 0.000 |
| Intestinal immune network for IgA production | 7 | 129.2 (112.5, 149.2) | 0.004 | 124.6 (108.4, 144.3) | 0.006 | 103.7 (102.4, 105.2) | 0.000 | 15.8 (8.8, 32.9) | 0.004 |
| HIF-1 signaling pathway | 6 | 129.6 (112.1, 149.0) | 0.000 | 124.9 (108.2, 143.8) | 0.002 | 103.7 (102.2, 105.2) | 0.000 | 15.7 (8.6, 33.6) | 0.000 |
| Viral protein interaction with cytokine and cytokine receptor | 11 | 129.5 (112.0, 149.8) | 0.002 | 124.9 (108.0, 145.2) | 0.002 | 103.7 (102.4, 105.1) | 0.000 | 15.5 (8.8, 34.4) | 0.002 |
| Adherens junction | 6 | 129.4 (112.5, 147.9) | 0.000 | 125.0 (108.7, 142.4) | 0.004 | 103.5 (102.4, 104.8) | 0.000 | 15.0 (8.7, 32.4) | 0.000 |
| Chemokine signaling pathway | 9 | 129.3 (114.2, 149.1) | 0.000 | 125.0 (110.2, 144.3) | 0.000 | 103.5 (102.1, 105.0) | 0.000 | 14.9 (8.1, 28.7) | 0.000 |
| Rheumatoid arthritis | 8 | 129.3 (113.0, 148.7) | 0.000 | 125.2 (109.5, 144.7) | 0.004 | 103.3 (102.0, 104.5) | 0.000 | 13.9 (7.8, 29.2) | 0.000 |
| Complement and coagulation cascades | 8 | 129.4 (111.8, 147.3) | 0.000 | 125.4 (108.3, 142.8) | 0.000 | 103.3 (101.9, 104.6) | 0.000 | 13.8 (7.7, 31.6) | 0.000 |
| Pathogenic Escherichia coli infection | 9 | 129.8 (112.6, 149.5) | 0.000 | 125.7 (109.1, 144.3) | 0.000 | 103.2 (101.3, 105.2) | 0.000 | 13.5 (5.0, 29.8) | 0.000 |
| African trypanosomiasis | 3 | 129.3 (113.0, 147.3) | 0.000 | 125.8 (109.6, 143.5) | 0.002 | 102.8 (101.7, 104.0) | 0.000 | 12.0 (6.4, 25.8) | 0.000 |
| NF-kappa B signaling pathway | 5 | 129.4 (112.6, 148.1) | 0.000 | 125.9 (109.4, 144.1) | 0.000 | 102.8 (101.7, 104.0) | 0.000 | 11.8 (6.3, 24.9) | 0.000 |
| Chagas disease | 3 | 129.3 (111.3, 148.2) | 0.000 | 125.9 (108.6, 145.0) | 0.002 | 102.7 (101.7, 103.8) | 0.000 | 11.5 (6.6, 25.0) | 0.000 |
| Cell adhesion molecules | 6 | 129.2 (111.9, 148.7) | 0.000 | 126.0 (109.2, 145.2) | 0.000 | 102.6 (101.7, 103.7) | 0.000 | 11.2 (6.3, 24.7) | 0.000 |
| Tuberculosis | 6 | 129.5 (113.0, 148.4) | 0.000 | 126.5 (110.2, 145.3) | 0.000 | 102.3 (101.1, 103.5) | 0.000 | 10.0 (4.4, 23.1) | 0.000 |
| ErbB signaling pathway | 3 | 129.9 (113.6, 148.1) | 0.000 | 126.9 (110.7, 144.5) | 0.000 | 102.4 (101.0, 103.8) | 0.000 | 10.0 (4.2, 21.8) | 0.000 |
| AGE-RAGE signaling pathway in diabetic complications | 4 | 129.4 (113.9, 148.9) | 0.000 | 126.5 (111.5, 145.7) | 0.002 | 102.2 (101.4, 103.2) | 0.000 | 9.6 (5.4, 19.9) | 0.000 |
| Malaria | 5 | 129.3 (113.0, 148.7) | 0.000 | 126.6 (110.3, 145.5) | 0.000 | 102.2 (101.2, 103.2) | 0.000 | 9.5 (5.1, 20.2) | 0.000 |
| IL-17 signaling pathway | 5 | 129.4 (112.9, 148.8) | 0.000 | 126.7 (109.9, 145.6) | 0.000 | 102.1 (101.2, 103.2) | 0.000 | 9.1 (4.6, 19.3) | 0.000 |
| Legionellosis | 3 | 129.4 (113.1, 146.5) | 0.000 | 126.9 (110.7, 143.7) | 0.000 | 102.0 (101.2, 102.8) | 0.000 | 8.6 (4.6, 18.7) | 0.000 |
| Focal adhesion | 6 | 129.4 (111.9, 149.7) | 0.000 | 126.8 (109.6, 146.2) | 0.000 | 102.0 (101.3, 102.9) | 0.000 | 8.6 (4.8, 19.3) | 0.000 |
| Th17 cell differentiation | 4 | 129.5 (112.9, 149.2) | 0.000 | 127.3 (110.8, 146.9) | 0.000 | 101.7 (100.7, 102.8) | 0.002 | 7.5 (2.9, 17.5) | 0.002 |
| Inflammatory bowel disease | 4 | 129.5 (113.5, 148.5) | 0.000 | 127.3 (111.7, 146.1) | 0.000 | 101.7 (101.0, 102.5) | 0.000 | 7.5 (3.8, 15.3) | 0.000 |
| Hematopoietic cell lineage | 3 | 129.5 (113.3, 148.1) | 0.000 | 127.3 (111.4, 145.9) | 0.000 | 101.7 (101.0, 102.5) | 0.000 | 7.4 (3.9, 15.4) | 0.000 |
| Lysosome | 6 | 129.2 (112.6, 148.3) | 0.000 | 127.1 (110.7, 146.0) | 0.000 | 101.6 (100.9, 102.5) | 0.000 | 7.0 (3.4, 15.8) | 0.000 |
| PD-L1 expression and PD-1 checkpoint pathway in cancer | 2 | 129.4 (112.2, 148.5) | 0.000 | 127.5 (110.9, 146.8) | 0.000 | 101.5 (100.5, 102.5) | 0.000 | 6.5 (2.1, 16.3) | 0.000 |
| ECM-receptor interaction | 4 | 129.5 (113.6, 148.3) | 0.000 | 127.6 (112.1, 146.2) | 0.000 | 101.5 (100.8, 102.2) | 0.000 | 6.4 (3.2, 12.9) | 0.000 |
| Regulation of actin cytoskeleton | 5 | 129.5 (113.0, 147.9) | 0.000 | 127.6 (111.1, 145.9) | 0.000 | 101.5 (100.9, 102.2) | 0.000 | 6.4 (3.4, 14.3) | 0.000 |
| Colorectal cancer | 2 | 129.8 (112.9, 147.8) | 0.000 | 128.2 (111.5, 146.0) | 0.000 | 101.3 (100.6, 101.9) | 0.000 | 5.4 (2.4, 12.0) | 0.000 |
| Pertussis | 1 | 129.4 (113.5, 149.4) | 0.000 | 128.0 (112.2, 147.9) | 0.000 | 101.1 (100.6, 101.7) | 0.000 | 4.7 (2.2, 9.7) | 0.000 |
| Leukocyte transendothelial migration | 2 | 129.8 (113.1, 148.8) | 0.000 | 128.4 (112.1, 147.2) | 0.000 | 101.1 (100.5, 101.7) | 0.000 | 4.7 (2.2, 10.3) | 0.000 |

Pathway-level mediation proportions were estimated by jointly modelling all positively mediating proteins within each pathway. All analyses were adjusted for age, sex, qualifications, BMI, Charlson Comorbidity Index (CCI), occupational benzene exposure, alcohol consumption, smoking history, moderate sleep, moderate physical activity, healthy diet score, proximity to major roads, and noise pollution. Statistical significance was assessed using two-sided Z-tests.

# **Supplementary Table 15. Pathway-level mediation analysis of GO biological process pathways based on positively mediating proteins.**

| **GO Biological Process pathways** | **N** | **Total effect** | | **Natural direct effect** | | **Natural indirect effect** | | **Proportion of mediation** | | |
| --- | --- | --- | --- | --- | --- | --- | --- | --- | --- | --- |
|  |  | **Estimate (95% CI)** | **P** | **Estimate (95% CI)** | **P** | **Estimate (95% CI)** | **P** | **Estimate (%, 95% CI)** | | **P** |
| Regulation of Cell Population Proliferation | 29 | 120.4 (104.7, 137.9) | 0.014 | 107.6 (105.4, 109.8) | 0.000 | 129.5 (112.3, 149.3) | 0.002 | 31.1 (18.5, 63.1) | | 0.002 |
| Positive Regulation of Cell Population Proliferation | 28 | 120.7 (105.2, 139.3) | 0.016 | 107.3 (105.1, 109.5) | 0.000 | 129.5 (112.9, 149.9) | 0.000 | 29.8 (18.5, 60.1) | | 0.000 |
| Positive Regulation of Cellular Process | 31 | 121.2 (104.8, 141.4) | 0.004 | 107.0 (104.4, 109.6) | 0.000 | 129.7 (112.3, 151.0) | 0.002 | 28.5 (15.9, 61.6) | | 0.002 |
| Positive Regulation of Apoptotic Process | 17 | 121.8 (105.6, 139.9) | 0.004 | 106.1 (103.8, 108.5) | 0.000 | 129.3 (112.1, 148.3) | 0.000 | 25.4 (14.1, 56.7) | | 0.000 |
| Positive Regulation of Programmed Cell Death | 12 | 121.9 (106.4, 139.6) | 0.002 | 106.1 (104.2, 108.0) | 0.000 | 129.3 (113.4, 148.7) | 0.000 | 25.4 (15.8, 51.8) | | 0.000 |
| Positive Regulation of Intracellular Signal Transduction | 19 | 121.8 (106.1, 140.3) | 0.006 | 105.7 (103.9, 107.5) | 0.000 | 128.8 (112.0, 147.3) | 0.000 | 24.1 (14.2, 50.9) | | 0.000 |
| Proteolysis | 9 | 122.3 (106.9, 141.8) | 0.002 | 105.8 (104.3, 107.4) | 0.000 | 129.3 (113.1, 149.0) | 0.000 | 24.0 (14.7, 49.6) | | 0.000 |
| Regulation of Apoptotic Process | 21 | 122.8 (107.4, 142.2) | 0.002 | 105.5 (103.3, 107.8) | 0.000 | 129.6 (113.4, 149.5) | 0.000 | 22.9 (12.6, 47.2) | | 0.000 |
| Cytokine-Mediated Signaling Pathway | 14 | 122.4 (106.8, 141.5) | 0.008 | 105.4 (103.7, 107.1) | 0.000 | 129.0 (112.4, 149.4) | 0.004 | 22.8 (13.6, 47.5) | | 0.004 |
| Positive Regulation of Multicellular Organismal Process | 11 | 122.8 (107.7, 139.7) | 0.002 | 105.3 (103.7, 107.0) | 0.000 | 129.3 (113.5, 147.5) | 0.000 | 22.2 (13.4, 44.4) | | 0.000 |
| Negative Regulation of Multicellular Organismal Process | 12 | 122.7 (107.7, 141.7) | 0.002 | 105.1 (103.5, 106.7) | 0.000 | 128.9 (113.6, 148.2) | 0.000 | 21.6 (13.0, 44.6) | | 0.000 |
| Regulation of Angiogenesis | 10 | 123.1 (106.5, 142.5) | 0.006 | 105.1 (103.8, 106.6) | 0.000 | 129.4 (112.0, 149.5) | 0.000 | 21.4 (13.1, 46.2) | | 0.000 |
| Positive Regulation of Cell Migration | 17 | 122.9 (106.9, 141.5) | 0.004 | 104.8 (102.7, 107.1) | 0.000 | 128.8 (112.1, 148.2) | 0.000 | 20.5 (10.7, 45.0) | | 0.000 |
| Regulation of Cell Migration | 19 | 123.2 (108.1, 141.5) | 0.002 | 104.7 (102.8, 106.3) | 0.000 | 129.0 (112.9, 148.2) | 0.000 | 19.9 (11.3, 39.4) | | 0.000 |
| + Reg of Phosphatidylinositol 3-Kinase/Prot Kinase B Signal Transduction | 8 | 123.1 (107.0, 140.9) | 0.002 | 104.5 (103.0, 106.0) | 0.000 | 128.6 (111.7, 147.3) | 0.000 | 19.2 (11.5, 41.9) | | 0.000 |
| Regulation of Phosphatidylinositol 3-Kinase/Protein Kinase B Signal Transduction | 8 | 123.1 (107.2, 141.9) | 0.004 | 104.5 (103.0, 106.1) | 0.000 | 128.6 (112.0, 147.7) | 0.000 | 19.2 (11.5, 41.4) | | 0.000 |
| Regulation of MAPK Cascade | 8 | 123.2 (108.0, 141.7) | 0.002 | 104.5 (103.3, 105.8) | 0.000 | 128.7 (112.9, 148.3) | 0.000 | 19.2 (11.9, 39.2) | | 0.000 |
| Positive Regulation of Cytokine Production | 12 | 123.6 (107.6, 141.1) | 0.004 | 104.5 (102.9, 106.2) | 0.000 | 129.2 (112.3, 147.0) | 0.000 | 19.2 (11.1, 41.9) | | 0.000 |
| Positive Regulation of Cell Motility | 13 | 123.4 (106.2, 141.8) | 0.002 | 104.5 (103.1, 106.1) | 0.000 | 128.9 (111.8, 147.9) | 0.000 | 19.1 (11.1, 44.9) | | 0.000 |
| Peptidyl-Tyrosine Phosphorylation | 4 | 123.9 (106.7, 142.5) | 0.006 | 104.3 (102.6, 106.2) | 0.000 | 129.2 (111.2, 148.2) | 0.000 | 18.2 (9.9, 41.8) | | 0.000 |
| Cellular Response to Transforming Growth Factor Beta Stimulus | 6 | 123.5 (107.7, 140.1) | 0.006 | 104.2 (103.0, 105.4) | 0.000 | 128.7 (112.3, 146.2) | 0.000 | 18.0 (11.4, 37.5) | | 0.000 |
| Negative Regulation of Cellular Process | 17 | 124.6 (108.7, 143.4) | 0.000 | 104.2 (102.6, 105.9) | 0.000 | 129.8 (113.5, 149.3) | 0.000 | 17.7 (9.5, 36.3) | | 0.000 |
| Cellular Response to Cytokine Stimulus | 8 | 124.5 (108.9, 144.8) | 0.002 | 104.2 (102.9, 105.5) | 0.000 | 129.7 (113.2, 150.9) | 0.000 | 17.6 (10.7, 34.6) | | 0.000 |
| Positive Regulation of MAPK Cascade | 12 | 123.8 (107.9, 142.7) | 0.000 | 104.0 (102.6, 105.3) | 0.000 | 128.8 (111.9, 148.2) | 0.000 | 17.2 (9.8, 35.9) | | 0.000 |
| Cell Surface Receptor Protein Tyrosine Kinase Signaling Pathway | 8 | 124.6 (108.5, 144.0) | 0.002 | 104.0 (102.9, 105.3) | 0.000 | 129.6 (113.2, 148.9) | 0.000 | 17.0 (10.3, 35.9) | | 0.000 |
| Transforming Growth Factor Beta Receptor Signaling Pathway | 5 | 123.8 (106.7, 142.5) | 0.002 | 103.9 (102.8, 105.1) | 0.000 | 128.7 (110.8, 147.8) | 0.002 | 16.9 (10.4, 37.8) | | 0.002 |
| Transforming Growth Factor Beta Receptor Superfamily Signaling Pathway | 5 | 123.8 (107.7, 142.3) | 0.004 | 103.9 (102.7, 105.0) | 0.000 | 128.7 (111.8, 147.7) | 0.000 | 16.9 (10.4, 35.5) | | 0.000 |
| Extracellular Matrix Organization | 10 | 124.8 (108.8, 143.1) | 0.002 | 104.0 (102.5, 105.8) | 0.000 | 129.8 (113.6, 148.7) | 0.002 | 16.8 (9.5, 35.4) | | 0.002 |
| Inflammatory Response | 11 | 124.4 (107.5, 141.8) | 0.000 | 103.9 (102.5, 105.4) | 0.000 | 129.2 (111.7, 147.2) | 0.000 | 16.7 (9.5, 37.6) | | 0.000 |
| #NAME? | 5 | 124.0 (106.7, 142.7) | 0.010 | 103.8 (102.6, 105.0) | 0.000 | 128.8 (110.5, 148.3) | 0.000 | 16.5 (10.1, 36.9) | | 0.000 |
| Negative Regulation of Cell Population Proliferation | 13 | 125.1 (109.2, 143.0) | 0.002 | 103.9 (102.5, 105.3) | 0.000 | 129.9 (113.5, 148.3) | 0.000 | 16.1 (9.1, 33.5) | | 0.000 |
| Regulation of Smooth Muscle Cell Proliferation | 6 | 124.6 (108.5, 143.4) | 0.000 | 103.8 (102.6, 105.1) | 0.000 | 129.3 (112.7, 148.9) | 0.000 | 16.1 (9.4, 35.7) | | 0.000 |
| Cell Surface Receptor Signaling Pathway via JAK-STAT | 4 | 124.6 (109.5, 143.8) | 0.000 | 103.7 (102.6, 105.0) | 0.000 | 129.2 (113.5, 148.7) | 0.000 | 15.7 (9.3, 31.7) | | 0.000 |
| Regulation of Peptidyl-Tyrosine Phosphorylation | 5 | 124.8 (108.6, 144.0) | 0.000 | 103.6 (102.4, 104.8) | 0.000 | 129.3 (112.2, 149.4) | 0.000 | 15.5 (9.1, 30.5) | | 0.000 |
| Negative Regulation of Apoptotic Process | 15 | 125.4 (109.5, 142.5) | 0.002 | 103.5 (101.4, 105.5) | 0.000 | 129.8 (113.3, 148.1) | 0.000 | 14.7 (5.9, 31.7) | | 0.000 |
| Positive Regulation of Epithelial Cell Proliferation | 9 | 125.6 (109.3, 143.8) | 0.004 | 103.5 (102.3, 104.8) | 0.000 | 130.0 (113.1, 148.7) | 0.000 | 14.7 (8.3, 29.6) | | 0.000 |
| Regulation of Inflammatory Response | 9 | 125.0 (109.2, 142.9) | 0.004 | 103.4 (102.0, 104.9) | 0.000 | 129.3 (112.7, 147.6) | 0.000 | 14.6 (7.9, 29.9) | | 0.000 |
| Wound Healing | 4 | 125.3 (108.0, 144.6) | 0.000 | 103.5 (102.4, 104.6) | 0.000 | 129.6 (111.6, 149.2) | 0.000 | 14.6 (8.4, 31.9) | | 0.000 |
| Positive Regulation of Protein Phosphorylation | 6 | 125.1 (108.2, 144.0) | 0.002 | 103.4 (102.1, 104.9) | 0.000 | 129.4 (111.7, 149.1) | 0.000 | 14.5 (8.1, 32.1) | | 0.000 |
| Cell-Cell Adhesion via Plasma-Membrane Adhesion Molecules | 4 | 125.4 (109.5, 143.2) | 0.004 | 103.4 (102.5, 104.5) | 0.000 | 129.7 (113.3, 148.0) | 0.002 | 14.5 (8.8, 28.2) | 0.002 | |
| Positive Regulation of Peptidyl-Tyrosine Phosphorylation | 6 | 125.0 (108.9, 144.2) | 0.000 | 103.4 (101.6, 105.2) | 0.000 | 129.2 (112.7, 148.8) | 0.000 | 14.5 (6.4, 31.4) | 0.000 | |
| Regulation of Immune Response | 4 | 125.1 (108.5, 144.1) | 0.000 | 103.4 (102.0, 104.9) | 0.000 | 129.3 (112.5, 148.0) | 0.000 | 14.4 (7.7, 33.9) | 0.000 | |
| Positive Regulation of Inflammatory Response | 9 | 125.2 (110.0, 144.4) | 0.002 | 103.4 (102.2, 104.6) | 0.000 | 129.4 (113.5, 148.6) | 0.000 | 14.3 (8.1, 28.5) | 0.000 | |
| Positive Regulation of Cell Differentiation | 15 | 125.7 (108.1, 145.3) | 0.002 | 103.4 (102.1, 104.7) | 0.000 | 129.9 (112.0, 150.2) | 0.000 | 14.2 (7.6, 32.7) | 0.000 | |
| Receptor-Mediated Endocytosis | 6 | 125.4 (109.1, 143.3) | 0.004 | 103.3 (102.2, 104.6) | 0.000 | 129.6 (113.4, 147.7) | 0.000 | 14.1 (8.3, 30.0) | 0.000 | |
| Intracellular Calcium Ion Homeostasis | 8 | 125.1 (109.1, 143.3) | 0.004 | 103.3 (102.1, 104.5) | 0.000 | 129.2 (112.4, 147.6) | 0.000 | 14.1 (7.9, 29.2) | 0.000 | |
| Extracellular Structure Organization | 5 | 125.7 (109.4, 145.8) | 0.002 | 103.3 (102.3, 104.5) | 0.000 | 129.8 (113.4, 150.4) | 0.000 | 14.0 (8.4, 28.0) | 0.000 | |

Pathway-level mediation proportions were estimated by jointly modelling all positively mediating proteins within each pathway. Only the top 45 of the 256 pathways ranked by mediation proportion are shown. All analyses were adjusted for age, sex, qualifications, BMI, Charlson Comorbidity Index (CCI), occupational benzene exposure, alcohol consumption, smoking history, moderate sleep, moderate physical activity, healthy diet score, proximity to major roads, and noise pollution. Statistical significance was assessed using two-sided Z-tests.
